# Gut microbiota signatures in cystic fibrosis: Loss of host CFTR function drives the microbiota enterophenotype

**DOI:** 10.1371/journal.pone.0208171

**Published:** 2018-12-06

**Authors:** Pamela Vernocchi, Federica Del Chierico, Alessandra Russo, Fabio Majo, Martina Rossitto, Mariacristina Valerio, Luca Casadei, Antonietta La Storia, Francesca De Filippis, Cristiano Rizzo, Cesare Manetti, Paola Paci, Danilo Ercolini, Federico Marini, Ersilia Vita Fiscarelli, Bruno Dallapiccola, Vincenzina Lucidi, Alfredo Miccheli, Lorenza Putignani

**Affiliations:** 1 Unit of Human Microbiome, Bambino Gesù Children's Hospital, IRCCS, Rome, Italy; 2 Cystic Fibrosis Unit, Bambino Gesù Children's Hospital, IRCCS, Rome, Italy; 3 Diagnostics of Cystic Fibrosis, Bambino Gesù Children's Hospital, IRCCS, Rome, Italy; 4 Department of Chemistry, Sapienza University of Rome, Rome, Italy; 5 Department of Agricultural Sciences, Division of Microbiology, University of Naples Federico II, Portici, Napoli, Italy; 6 Division of Metabolism, Bambino Gesù Children's Hospital, IRCCS, Rome, Italy; 7 Department of Environmental Biology; Sapienza University of Rome, Rome, Italy; 8 CNR-Institute for Systems Analysis and Computer Science (IASI), Rome, Italy; 9 Scientific Directorate, Bambino Gesù Children's Hospital, IRCCS, Rome, Italy; 10 Unit of Parasitology Bambino Gesù Children's Hospital, IRCCS, Rome, Italy; Institut Pasteur, FRANCE

## Abstract

**Background:**

Cystic fibrosis (CF) is a disorder affecting the respiratory, digestive, reproductive systems and sweat glands. This lethal hereditary disease has known or suspected links to the dysbiosis gut microbiota. High-throughput meta-omics-based approaches may assist in unveiling this complex network of symbiosis modifications.

**Objectives:**

The aim of this study was to provide a predictive and functional model of the gut microbiota enterophenotype of pediatric patients affected by CF under clinical stability.

**Methods:**

Thirty-one fecal samples were collected from CF patients and healthy children (HC) (age range, 1–6 years) and analysed using targeted-metagenomics and metabolomics to characterize the ecology and metabolism of CF-linked gut microbiota. The multidimensional data were low fused and processed by chemometric classification analysis.

**Results:**

The fused metagenomics and metabolomics based gut microbiota profile was characterized by a high abundance of *Propionibacterium*, *Staphylococcus* and Clostridiaceae, including *Clostridium difficile*, and a low abundance of *Eggerthella*, *Eubacterium*, *Ruminococcus*, *Dorea*, *Faecalibacterium prausnitzii*, and Lachnospiraceae, associated with overexpression of 4-aminobutyrate (GABA), choline, ethanol, propylbutyrate, and pyridine and low levels of sarcosine, 4-methylphenol, uracil, glucose, acetate, phenol, benzaldehyde, and methylacetate. The CF gut microbiota pattern revealed an enterophenotype intrinsically linked to disease, regardless of age, and with dysbiosis uninduced by reduced pancreatic function and only partially related to oral antibiotic administration or lung colonization/infection.

**Conclusions:**

All together, the results obtained suggest that the gut microbiota enterophenotypes of CF, together with endogenous and bacterial CF biomarkers, are direct expression of functional alterations at the intestinal level. Hence, it’s possible to infer that CFTR impairment causes the gut ecosystem imbalance.This new understanding of CF host-gut microbiota interactions may be helpful to rationalize novel clinical interventions to improve the affected children’s nutritional status and intestinal function.

## Background

Cystic fibrosis (CF) is the most common autosomal recessive disease among Caucasians, affecting more than 30,000 individuals in the United States and more than 85,000 individuals worldwide [1,2]. Within the context of the host genetic variability [3,4], determined by cystic fibrosis transmembrane conductance regulator (*CFTR)* gene mutation, the phenotypic variability is due to the wide variability of the *CFTR* gene mutations (>2000) [1]. Abnormal expression of the *CFTR* gene, which encodes a protein involved in the transport of chloride ions across cell membranes, leads to dysregulation of epithelial fluid transport in the lungs, gut, pancreas, liver and other organs [5]. This dysregulation results in ionic imbalance, with a greater Cl^-^ and water loss in the sweat glands, respiratory infections, and chronic pulmonary and intestinal inflammation caused by secretion thickening due to the reduced diffusion of Cl^-^ ion and water in CF patients [6]. Ionic imbalance disregulates intestinal homeostasis and leads to gut microbiota dysbiosis. Moreover, at the intestinal level, a reduced secretion of bicarbonate induces the pH reduction. Altered microbial ecosystem impairs the adsorption of nutrients as well as host immunity and supports the growth of potentially pathogenic microbes [5]. Antibiotic therapy for controlling CF chronic lung colonization/infections may result in the decrease or even loss of bacterial groups from the gut microbiota “ecosystem” [3]. The investigation of gut microbiota metacommunities and their functions by targeted-metagenomics has allowed the study of CF gut microbiota enterogradients and the assessment of their modulation and interactions with external *stimuli* and with the lung microbiota. Moreover, the complex metabolite network, produced by the gut microbial-host co-metabolism, displays distinct “metabotypes” [7,8], which contributes to the resolution of disease-driven shifts in host-microbiota interplay [9,10].

By using multidimensional metagenomics and metabolomics data in a systems medicine framework, we found that the gut microbiota “milieu”, in a pediatric CF cohort in clinical stability, is primarily caused by host CFTR function impairment and to a lesser extent to patients’ age, disease phenotype, lung microbiota, and chronic antibiotic regimen, and other external *stimuli*. Metagenomics and metabolomics multidimensional data, after reduction and fusion as low data, produced a microbiota-based predictive and functional model, which strongly suggested microbiota signatures driven by the altered CFTR functions.

## Results

### Phenomics of patients with CF and healthy children

The genotypes of 24 of 31 patients (77%) indicated full-blown expression of the disease, including pancreatic insufficiency (PI). Meconium ileus was confirmed in 9 of 31 CF patients (26%). The median value of Z-score (weight/length, W/L of <2 years of age) or body mass index (BMI) (>2 years of age) was −0.1 (range, −1.5–3.2) (kg/m^2^). Sweat chloride test results of the patient cohort ranged from 60 to 140 mmol/L (average value, 92.57 mmol/L). The **S1 and S2 Tables** report all phenomic data for CF patients and healthy children (HC).

CF patients were categorized on the basis of chronic antibiotic regimen, as follows: *i*) no antibiotic therapy (NA, 17/31 patients); *ii*) in antibiotic therapy (AT, 14/31), subclassified as antibiotic therapy for aerosol (AA, 7/31) or azithromycin plus antibiotic therapy for aerosol (A+AA, 7/31) (**S1** and **S3 Tables**). Amongst the 31 CF patients, 17 underwent exacerbation regimen (ER) following pulmonary symptoms: the therapy was additive to chronic regimen for 10/17 and alternative for 7/17 patients **(S3 Table)**.

### Culturomics of hypopharyngeal secretions

Aerobic and microaerophilic bacterial species, more commonly encountered in the upper airways, have been detected in significant numbers in CF hypopharyngeal secretions.

The microbial species detected by culturomics from the hypopharyngeal secretions of CF patients were in agreement with those recently described in by Cribbs and Beck [11].

The lung microbiota (LM) was classified into 3 main groups, based onto evidences described in literature about the microrganism’s pathogenic role [12]: i) populated by viridans bacteria and/or *Escherichia coli*, *Haemophilus influenzae*, *Serratia marcescens*, *Enterobacter cloacae*, *Branhamella catarrhalis* and *Streptococcus pneumoniae* (LM1, 11/31 patients); ii) populated by methicillin susceptible and methicillin resistant *Staphylococcus aureus* (MSSA and MRSA respectively), *Stenotrophomonas maltophilia*, *Eikenella corrodens*, *Acinetobacter* spp., *Flavobacterium meningosepticum*, *Achromobacter xylosoxidans*, *Candida parapsilosis*, *Klebsiella oxytoca* and *Acinetobacter lwoffii* (LM2, 15/31); iii) mainly colonized by *Pseudomonas aeruginosa* (LM3, 5/31) (**S1 Table**).

Specifically, 26 of 31 patients (84%) had the following microbial respiratory tract infections/colonizations: 14 of 31 (45%) with *S*. *aureus* (2 with methicillin-resistant *Staphylococcus aureus* (MRSA)); 5 (16%) with *P*. *aeruginosa*; 2 (6%) with *S*. *malthophilia*; 7 (22.6%) with *E*. *coli* (4 with *E*. *coli* ESBL+); 9 (29%) with *H*. *influenzae*; 3 (9.6%) with *E*. *cloacae*, *S*. *pneumoniae*, or *B*. *catarrhalis*; 1 (3%) with *C*. *parapsilosis*, *K*. *oxytoca*, *A*. *xylosoxidans*, *F*. *meningosepticum*, *Acinetobacter* spp., *E*. *corrodens*, or *A*. *lwoffii*. Five of 31 patients (16%) did not show any infection/colonization (**S1 Table**).

### Targeted- metagenomics and metabolomics profiling of gut microbiota

After data filtering, a total of 316,006 sequence reads of 16S rRNA gene amplicons were obtained with an average of 5,356 reads/sample and an average length of 487 bp (calculated after primer removal). Resulting operational taxnomic units (OTUs) were obtained by sequence-database matching (**S4 Table**). Genus-level comparisons were performed on a reduced matrix of 31 of 165 total variables. Criteria for inclusion of variables were a value different from 0 in ≥80% of the total sample set. Variables that were present only in 1 class were included (**S4 Table**).

**Fig 1:**
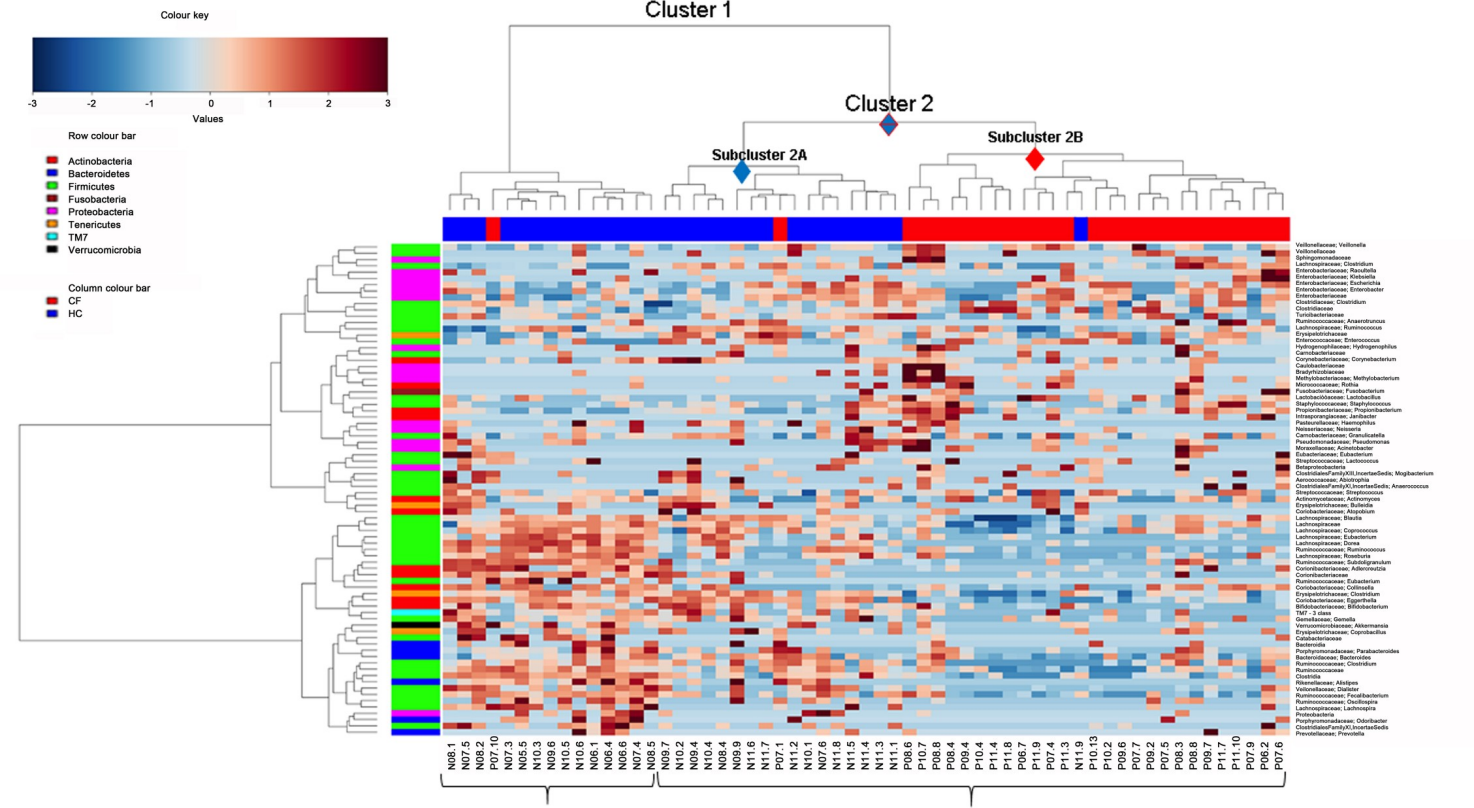
Hierarchical clustering of CF patients and HC subjects according to OTU distribution at the genus level. In the heatplot, hierarchical ward-linkage clustering is based on the Spearman’s correlation coefficient of OTU levels. The color scale represents the scaled level of each variable: red, high level; blue, low level. The column bar is colored according to the subject category. The row bar is colored according to the phylum level taxonomy.

The distribution of OTU levels at the genus level in the CF and HC groups is reported in **Fig 1**.

The disease phenotype (CF vs. HC) significantly influenced microbiota phylotypes, as measured by using ADONIS and ANOSIM (p≤0.001). Results of beta-diversity highlighted a clear separation between CF and HC groups, showing that the disease phenotype significantly influenced microbiota phylotypes (**S1 Fig**). Hierarchical cluster analysis revealed two main clusters, 1 and 2, the latter being split into two subclusters (2A and 2B) (**Fig 1**). Cluster 1 included 15 older HC (3–6 years) subjects, while the younger HC (1–2 years) were incorporated into the subcluster 2A. OTU levels in all HC subjects were distributed, as expected, in an age-dependent manner. Subcluster 2B included all CF patients who exhibited a different OTU level distribution, regardless of age (**Fig 1**).

In particular, hierarchical clustering analysis (**Fig 1**) showed that HC were characterized for overabundant OTUs as follows: *Prevotella*, Clostridiales family XI Incertae Sedis, *Odoribacter*, Proteobacteria, *Lachnospira*, *Oscillospira*, *Faecalibacterium*, *Dialister*, *Alistipes*, Clostridia, Ruminococcaceae, *Clostridium* (Ruminococcaeae), *Bacteroides*, *Parabacteroides*, *Bacteroidia*, *Coprobacillus*, *Akkermansia*, *Gemella*, *Bifidobacterium*, TM7, *Atopodium*, *Bulleidia*, *Actinomyces*, *Blautia*, *Coprococcus* (Lachnospiraceae), *Clostridium* (Erysipelotrichaceae), *Colinsella*, *Eubacterium* (Lachnospiraceae), Coriobacteriaceae, *Subdoligranulum*, *Egghertella*, *Roseburia*, *Dorea*, *Ruminococcus*, and *Eubacterium* (Ruminococcaceae). CF patients had a higher level of the following OTUs: *Streptococccus*, *Acinetobacter*, *Lactococcus*, *Janibacter*, *Propionibacterium*, *Staphylococcus*, *Lactobacillus*, *Fusobacterium*, *Rothia*, *Corynebacterium*, Corionobacteriaceae, *Enterococcus*, Erysipelotrichaceae, *Ruminococcus* (Lachnospiraceae), Turicibacteriaceae, Clostridiaceae, *Clostridium* (Clostridiaceae), *Enterobacter*, *Escherichia*, *Klebsiella*, *Raoultella*, *Clostridium* (Lachnospiraceae), *Sphingomonas*, Veillonellaceae, *Veillonella*, Enterobacteriaceae, *Pseudomonas*, *Granulicatella*, *Neisseria*, *Haemophilus*, *Methylbacterium*, Bradyrhizobiaceae, Caulobacteriaceae, and *Hydrogenophilus*.

To assess microbial community structures in patients with CF vs. HC, we calculated measures of alpha-diversity with the Chao1 index, which summarizes the microbial diversity within each sample (**S5 Table**). The Shapiro-Wilk test was performed on Chao1 indices for each analyzed group and returned a normal distribution to each data set (data not shown).

**Fig 2:**
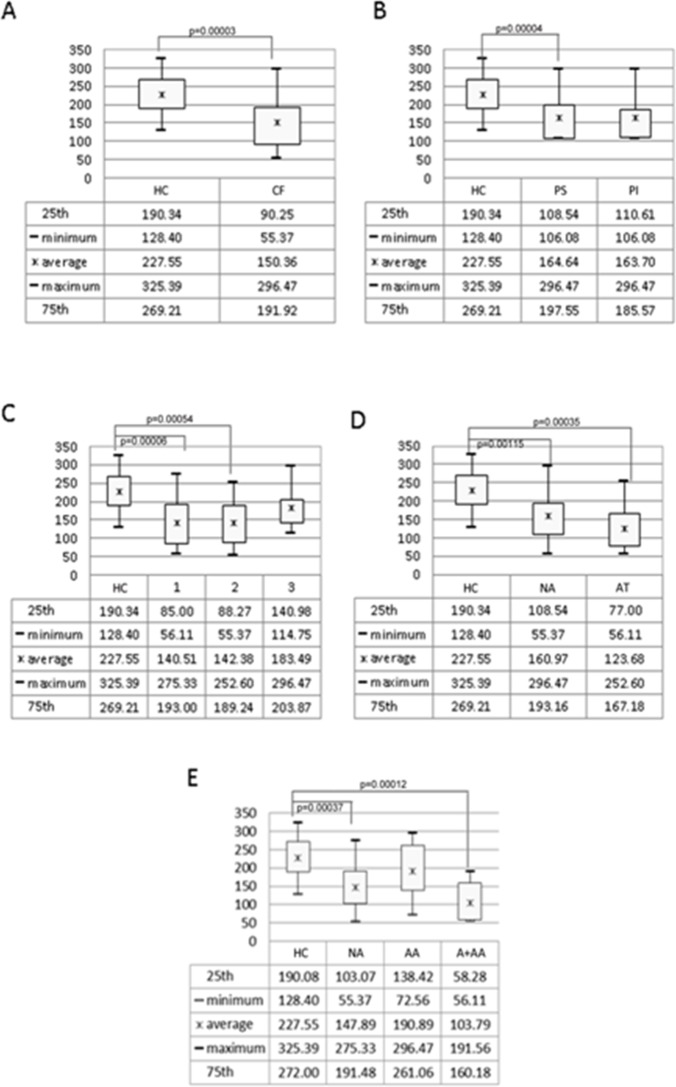
Box plots of the average values of Chao1 index for each CF/HC group. The plot reports the average, minimum (min), maximum (max) values, 25^th^, and 75^th^ quartiles calculated for HC, CF groups (**A**); HC, PS, and PI groups (**B**); HC, 1 (CF: populated by viridans bacteria *Haemophilus influenzae*, *Streptococcus pneumoniae*, *Escherichia coli*, *E*. *coli* ESBL+, *Serratia marcescens*, *Enterobacter cloacae*, and *Brahnamella catarrhalis*); 2 (CF: *Sthapylococcus aureus*, MRSA, *Stenotrophomonas maltophilia*, *Eikenella corrodens*, *Acinetobacter* spp., and *Flavobacterium meningosepticum)*; 3 (CF: *Pseudomonas aeruginosa* and *Pseudomonas* spp.) groups (**C**); HC, NA, AT groups (**D**); and HC, NA, AA, and A+AA groups (**E**). The summary tables show detailed values. P-values reaching the level of significance are reported on the respective box plot.

The Chao1 index was significantly different for CF and HC subjects (p = 0.00003) (**Fig 2A**), with a much lower value of alpha-diversity for CF compared to HC subjects. The Chao1 index, calculated in relation to pancreatic function (pancreatic insufficiency, PI and pancreatic sufficiency, PS) revealed a statistically significant difference for PS patients and HC (p = 0.00004), with a decresing diversity level trend from HC to PI (**Fig 2B**). The gut microbiota diversity level, calculated in respect to lung microbiota groups, was significantly different for LM1 (p = 0.00006) and LM2 (p = 0.00054), compared to HC, with a slight decrease for LM3 vs. HC (**Fig 2C**). Also, diversity level of patients receiving no antibiotic therapy (NA) and antibiotic therapy (AT) under chronic regimen vs. HC was significantly different (p = 0.00115 and p = 0.00035, respectively), with a decreasing value scale from HC to AT (**Fig 2D**). A statistically significant difference was observed as well for the azithromycin (A) plus antibiotic therapy for aerosol (A+AA) subclass vs. HC (p = 0.00012) and for NA vs HC (p = 0.00037). The diversity level of the antibiotic therapy for aerosol (AA) subgroup was intermediate between NA and A+AA (**Fig 2E**).

Bar charts of OTUs relative abundances in HC and CF reported phyla (A), families (B), and genus/species (C) in **S2 Fig.**

Comparison of OTU relative abundance between the HC and CF groups at OTUs level, performed by Kruskal-Wallis test, was plotted in **S3 Fig** and summarized in **Table 1**.

**Table 1:** Comparison of OTUs relative abundance between HC and CF entire phenotype; HC vs. antibiotic therapy- and pancreatic function -depending CF phenotypes (PI vs. PS) at species levels.

DISEASE PHENOTYPE: ENTIRE CF CATEGORY						
**Ranking phyla**	**Ranking family**	**HC**	**CF**		**p value**	**FDR**
Firmicutes	Lachnospiraceae	1.53	0.69		0.015	0.096
Firmicutes	Clostridiaceae	0.88	9.83		0.017	0.104
**Ranking phyla**	**Ranking species**	**HC**	**CF**		**p value**	**FDR**
Firmicutes	Eubacterium	0.53	0.03		0.000	0.019
Firmicutes	*Dorea formicigenerans*	0.68	0.02		0.000	0.017
Actinobacteria	*Eggerthella lenta*	2.00	0.24		0.001	0.025
Firmicutes	Ruminococcaceae *Clostridium*	0.31	0.03		0.001	0.031
Firmicutes	*Faecalibacterium*	0.07	0.00		0.002	0.036
Firmicutes	*Clostridium* sp., SS2/1	2.52	0.27		0.003	0.045
Tenericutes	Erysipelotrichaceae	6.77	0.23		0.004	0.052
Firmicutes	*Parvimonas micra*	0.02	0.00		0.005	0.059
Tenericutes	*Solobacterium moorei*	0.04	0.01		0.007	0.072
Firmicutes	*Staphylococcus*	0.00	0.04		0.008	0.082
Actinobacteria	*Propionibacterium acnes*	0.08	0.43		0.009	0.077
Firmicutes	*Faecalibacterium prausnitzii*	0.23	0.00		0.011	0.092
Firmicutes	*Clostridium difficile*	0.00	2.90		0.014	0.099
DISEASE PHENOTYPE: ANTIBIOTIC THERAPY-DEPENDING CF CATEGORY						
**Ranking phyla**	**Ranking species**	**HC**	**NA**^**1**^	**AT**	**p value**	**FDR**
Firmicutes	*Eubacterium*	0.53	0.01	0.04	0.001	0.100
Firmicutes	*Dorea formicigenerans*	0.68	0.00	0.04	0.002	0.085
Actinobacteria	*Eggerthella lenta*	2.00	0.07	0.37	0.003	0.103
DISEASE PHENOTYPE: PANCREATIC FUNCTION-DEPENDING CF CATEGORY						
**Ranking phyla**	**Ranking species**	**HC**	**PI**	**PS**	**p value**	**FDR**
Firmicutes	*Eubacterium*	0.53	0.02	0.06	0.001	0.100
Firmicutes	*Dorea formicigenerans*	0.68	0.00	0.10	0.002	0.082
Actinobacteria	*Eggerthella lenta*	2.00	0.14	0.60	0.003	0.104

^1^Legend: NA: no antibiotic therapy; AT, antibiotic therapy for chronic regimen; PS: pancreatic sufficiency; PI: pancreatic insufficiency. The non-parametric data were analysed with Kruskal–Wallis one-way analysis of variance (ANOVA) by ranks. p values and FDR adjusted p value were reported. A level of *P*<0.05 with a FDR~0.1 was accepted as statistically significant. Data are presented as means. Only statistically significant differences are reported.

At family level Erysiperlotrichaceae and Lachnospiraceae were abundant in HC respect to CF (**Table 1**).

Moreover, at genus/species level *Eubacterium*, *Dorea formicigenerans*, *Eggerthella lenta*, *Clostridium* (Ruminococcaceae), *Faecalibacterium*, *Clostridium* sp., SS2/1, *Parviromonas micra*, *Solobacterium moorei*, *Faecalibacterium prausnitzii*, were more abundant in HC compared to CF patients (FDR adjusted p value ≤0.1), while *Staphylococcus*, *Propionibacterium acnes*, *Clostridium difficile* and Clostridiaceae were higher in CF (FDR adjusted p value ≤0.1). *C*. *difficile* was slightly higher in patients (**Table 1**). Pairwise tests reveled that *E*. *lenta* was significantly increased in HC respect to each of the NA, A+AA and AA subsets (data not shown), while pancreatic function categories showed that *D*. *formicigerans* and *E*. *lenta* were statistically higher in HC only with respect to PI while *Eubacterium* appeared increased in HC compared to both PI and PS (data not shown).

To ascertain the significance of the comparison between OTU distributions in groups subjected to antibiotic therapy (A+AA and AA), under chronic regimen, the Mann-Whitney test was performed **(S6 Table)**. Significantly higher abundance of *Clostridium*, *Clostridium hiranosis*, *Eubacterium*, and *Faecalibacterium* were found in CF patients who received AA therapy (**S6 Table).** Concerning exacerbation regimen, underwent by only 17/31 patient, the treatment interruption for at least 2 weeks before stool collection for microbiome analyses, did not seem to induce a marked modification of the OTU distributions, when compared to gut microbiota profile not associated to exacerbation regimen (data not shown).

### Metabolomics profiling of gut microbial communities

#### Volatile organic (VOCs) and non volatile compounds profiling

Two-hundred and sixteen VOCs for all CF and HC subjects were identified, quantified, and grouped into 17 chemical classes by gas chromatography–mass spectrometry solid phase microextraction (GC-MS/SPME): alcohols (n 44); alkenes (n 8); alkanes (n 14); ketones (n 29); esters (n 43); acids (n 16); phenols (n 5); sulfur compounds (n 2); aldehydes (n 20); furanones (n 2); eterocycles (n 3); indoles (n 4); terpenes (n 20); pyridine (n 2); pyrimidine (n 1); furans (n 1); and piperidine (n 2). High variability was found among subjects, while technical replicates showed low variability (**S7 Table**). Patients with CF had slightly higher total median values of alcohols, in particular ethanol (≥2-fold that of HC) (p = 0.03) and 1-propanol (p = 0.047), compared to HC fecal samples. The total and median values of esters was higher among CF children than among HC, in particular for propylbutyrate (p = 0.057). The total values of alkanes was higher among patients with CF than among HC, especially for dodecane (p = 0.051). The total value of aliphatic and aromatic aldehydes was higher among HC than CF patients, especially for heptanal (p = 0.006), hexanal (p = 0.007), nonanal (p = 0.007), and octanal (p = 0.025). The total and median amounts of ketones were higher in the HC group, but some molecules (*e*.*g*., 2-pentanone [p = 0.034], 2,4-octanedione [p = 0.024]) were much higher in HC relative to CF patients. Total and median values of phenols were higher in HC than in the CF group, especially for phenol (p = 0.013) and 4-methylphenol *(p*-cresol); in contrast, 2,6-bis-(1,1-dimethylethyl)-phenol (p = 0.022) levels were higher in CF samples. The total amounts of furanones -in particular dihydro-2-methyl-3(2H)-furanone (p = 0.000) were higher in HC (**S7 Table**).

Twenty-two non volatile metabolites were detected, differentially assigned (*i*.*e*, acids, amino acids, amines, and sugars) and quantified by proton nuclear magnetic resonance (^1^H-NMR) for CF and HC samples (**S8 Table**). Short-chain fatty acids, such as acetate (p = 0.0032), butyrate (p = 0.0032), and propionate (p = 0.006) were higher among HC (**S8 Table**). Amino acids, such as isoleucine (p = 0.006), leucine (p = 0.037), alanine (p = 0.011), tyrosine (p = 0.011), asparagine (p = 0.011), glutamic acid (p = 0.0003), sarcosine (p = 0.00002), and uracil (p = 0.001), were elevated in the HC group vs. the CF group (**S8 Table**). In contrast, 4-aminobutyrate (GABA) (p = 0.039) and choline (p = 0.037) were higher among CF patients than among HC (**S8 Table**).

### Predictive model of CF-associated gut microbiota based on reduction of big multidimensional to smart -omics data

#### Matrix reduction of operational taxonomic units

Genera-level comparisons were performed on a reduced matrix of variables. Inclusion criteria for variables were measured values different from 0 in ≥80% of the total sample and variables exclusively present in a group. The reduced genus matrix included the following 31 of 165 variables: 1) *Actinomyces*; 2) Bacteroides; 3) *Bifidobacterium*; 4) *Blautia*; 5) Clostridiaceae; 6) *Clostridium*; 7) *Collinsella*; 8) *Coprococcus*; 9) *Corynebacterium*; 10) *Eggerthella*; 11) *Enterobacter*; 12) Enterobacteriaceae; 13) *Enterococcus*; 14) Erysipelotrichaceae; 15) *Escherichia*; 16) *Eubacterium*; 17) *Faecalibacterium*; 18) *Dorea*; 19) *Dialister*; 20) *Gemella*; 21) *Granulicatella*; 22) Lachnospiraceae; 23) *Lactobacillus*; 24) *Propionibacterium*; 25) *Roseburia*; 26) Ruminococcaceae; 27) *Ruminococcus*; 28) *Streptococcus*; 29) Turicibacteraceae; 30) *Veillonella*; 31) others.

#### Metabolomic profiling with gas–chromatographic-mass spectrometry/solid-phase microextraction

Statistical significance of the partial least squares discriminant analysis (PLS-DA) model from GC-MS/SPME data was ascertained by NMC and AUROC (both p<0.001) but not by DQ^2^ (p = 0.46) (**S4 Fig**). The model built on probabilistic quotient normalization (PQN) and Pareto-scaled data was represented by 5 significant latent variables (**S4A Fig**). Variables Importance in Projection (VIP)>2 (**S4B Fig**) included lower levels (pale blue) of 4-methylphenol (p-cresol), indole, phenol, methylacetate, butyric acid, pentanoic acid, caproic acid, 2-pentanone, 2,3-butanedione, 3-methylpentanoic acid, 4-methylpentanoic acid, and 4-methylpentanone. Higher levels (dark blue) of ethanol, propylbutyrate, 1-propanol, pyridine, and 3-methyphenol were detected in patients with CF vs. HC. The total correct classification rate (ccr) was 74.0% ± 4.8% (ccr, 65.1% ± 7.4% and 82.0% ± 5.3% for HC and CF, respectively) (**S4C Fig**).

#### Metabolomic profiling with proton nuclear magnetic resonance spectroscopy

**S5 Fig** depicts a plot of PLS-LV scores (**S5A Fig**), VIP (**S5B Fig**), and figures of merit (**S5C Fig**) of ^1^H-NMR analysis. Statistical significance of the PLS-DA model was achieved by NMC, AUROC, and DQ^2^ (p<0.001). The model built on autoscaled data included 3 significant LVs. The total correct classification rate was 81.0% ± 3.7% (79.5% ± 5.0% and 82.4% ± 4.7% for HC and CF, respectively). VIPs were diminished (pale red) for sarcosine, uracil, glucose, and glutamate and were higher (dark red) for 4-aminobutyrate (GABA) and choline.

#### Multivariate model of the CF gut microbial ecosystem

**Fig 3:**
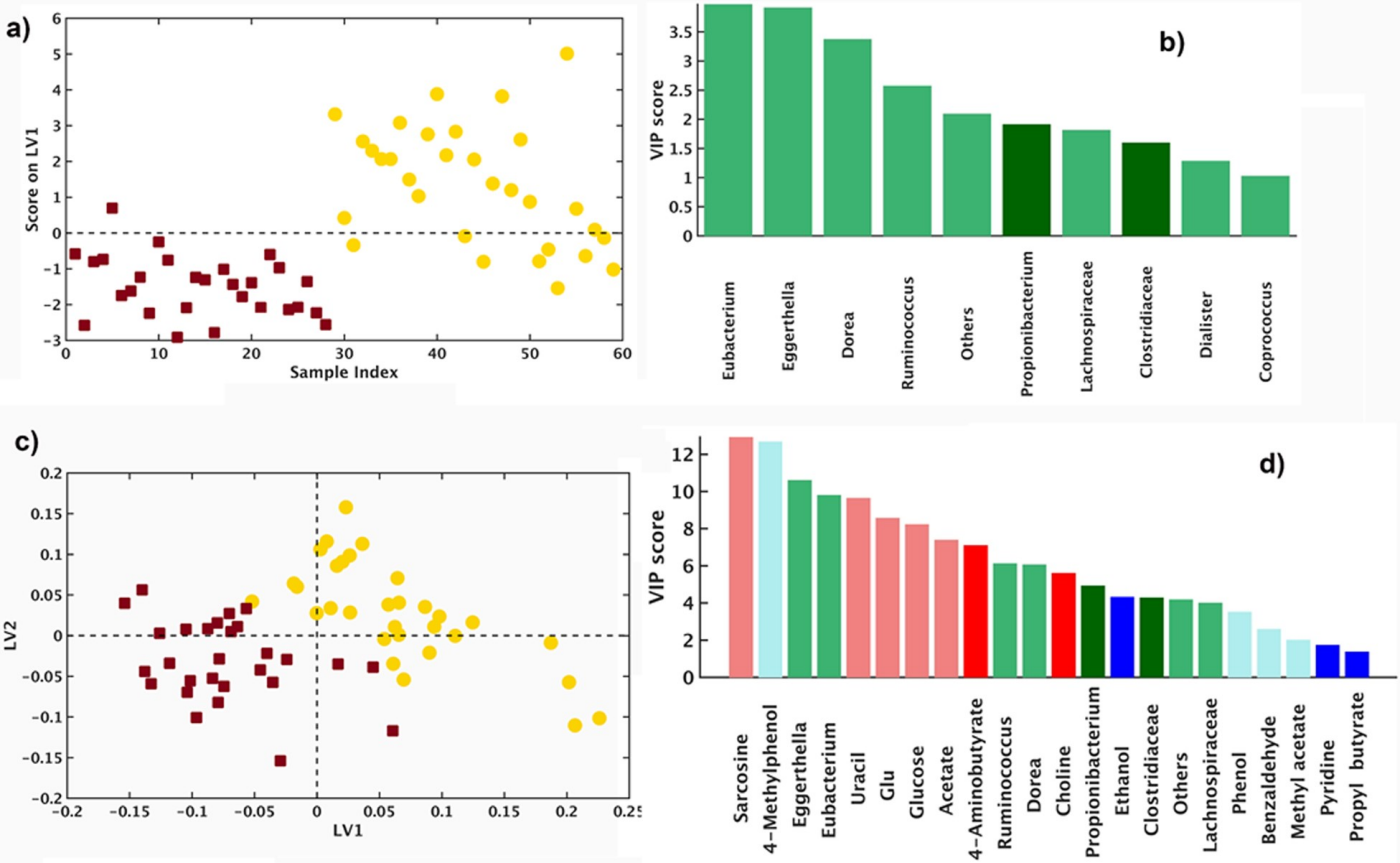
PLS-DA results. **OTU (genus level) reduced analysis**. Panel **A**. LV scores plot: yellow, HC; dark red, CF. Panel **B**. histograms representing VIP values: pale green, low levels; dark green, high levels in CF. Panel **C**. Low data fused: OTUs (genus level), GC-MS/SPME, and ^1^H-NMR analysis. LVs score plot: yellow, HC; dark red, CF. Panel **D**. histograms representing VIP values: pale blue, pale red, pale green, low levels; dark blue, dark red, dark green, high levels in CF.

The multivariate data analysis strategy performed by PLS-DA, first on the initial data matrices obtained from separate investigation approaches, namely target-metagenomics (**Fig 3A and 3B**), GC-MS (**S4A–S4C Fig**) and ^1^H-NMR-based (**S5A–S5C Fig**) metabolomics, and then, on the whole set of integrated data obtained from the above meta-omic platforms (*i*.*e*., low level fused data matrix), was exploited to characterize the gut microbial ecosystem of CF children (**Fig 3C and 3D**).

**Fig 3** displays the PLS Latent Variables (LVs) scores plot (**Fig 3A**) and the VIP (**Fig 3B**) on each OTU (at the genus level). The statistical significance of the PLS-DA model was characterized by number of misclassifications (NMC) and Area Under the Receiver Operating Characteristic (AUROC) (both p<0.001) and by Discriminant Q^2^ (DQ^2^) (p = 0.04). The model built on autoscaled data was represented by one significant LV1, 12.4%. The total correct classification rate was 78.4 ± 3.0% (87.9 ± 5.4 and 70.0 ± 3.9% for HC and CF, respectively). VIP>1.5 were lower abundances (pale green) of *Eubacterium*, *Eggerthella*, *Dorea*, *Ruminococcus*, Lachnospiraceae, *Dialister*, and *Coprococcus* and higher abundances (dark green) of *Propionibacterium* and Clostridiaceae in CF patients as compared to HC. OTUs, VOCs, and ^1^H-NMR data were filled into a single data block. In low level data fusion, individual data blocks were concatenated to form a single, augmented data matrix, to be used for classification modeling by means of PLS-DA. The model built on block scaled data was represented by three significant LVs. The LVs score plot of HC compared to CF is reported in **Fig 3C** and the LV score plot in **Fig 3D**. The PLS-DA model reached statistical significance for NMC/AUROC (p<0.001) and for DQ^2^ (p<0.005). The total correct classification rate was 85.4±3.4% (86.1±4.8 and 84.8±6.1 for HC and CF, respectively). VIP included lower level of sarcosine, 4-methylphenol, *Eggerthella*, *Eubacterium*, uracil, glucose, glutamate, *Ruminococcus*, *Dorea*, Lachnospiraceae, phenol, benzaldehyde, and methylacetate and a higher level of *Propionibacterium*, ethanol, Clostridiaceae, 1-propanol, 2,3-butanedione, GABA, choline, propylbutyrate, and pyridine (**Fig 3D**).

### Spearman rank test of targeted metagenomic and metabolomic data

The Spearman rank test was applied to assess the relationship between targeted metagenomics and metabolomics variables (**S9** and **S10 Tables**).

#### Metagenomics

In particular, in the correlation analysis between OTUs (species level), *C*. *difficile* was positively correlated with *Propionibacterium* spp., such as *P*. *acnes* (R = 0.410, p = 0.001) and *P*. *granulosum* (R = 0.307, p = 0.018). *C*. *difficile* was negatively correlated with Lachnospiraceae (R = −0.301, p = 0.016), *E*. *lenta* (R = −0.345, p = 0.007), and *F*. *prausnitzii* (R = −0.451, p<0.001) (**S9 Table)**.

#### GC-MS/SPME

The following correlations between OTUs and VOCs were found: *i*) negative correlations (genus-level) with *Dorea* and *Eggerthella* for 1-propanol, ethanol, ethyl acetate, heptanal, hexanal, nonanal, octanal, propyl butyrate, and treatadecane (all p≤0.05) (**S9 Table**). Similarly, at the species level, *D*. *formicigerans* and *E*. *lenta* were significantly negatively (p≤0.05) correlated with 1-propanol, ethanol, ethyl butyrate, heptanal, hexanal, nonanal, octanal, propyl butyrate, and treatadecane. At the genus level, Clostridiaceae, *Propionibacterium*, and *Pediococcus* all were positively correlated with most detected molecules, including alcohols (*i*.*e*., ethanol, 1-propanol, 2-octanol), esters (*i*.*e*., ethyl acetate, ethyl butyrate, propyl butyrate), aldehydes (*i*.*e*., heptanal, hexanal, octanal), phenols (*i*.*e*., 4-methylphenol), and pyridine; however, only *Pediococcus* and *Propionibacterium* had statistically significant correlations (p≤0.05). Clostridiaceae, in particular *C*. *difficile*, was significantly positively correlated (p≤0.05) with 1-propanol, ethanol, ethyl acetate, ethyl propionate, hexanal, and propyl butyrate (**S9 Table**).

#### ^1^H-NMR

The following major correlations between metabolites and OTUs (genus-level) were detected by ^1^H-NMR (**S10 Table**): i) positive correlations (not statistically significant, p≥0.05) between *Acinetobacter*, *Bulleidia*, Clostridiaceae, *Pediococcus*, and *Propionibacterium* with GABA, choline and acetate; ii) negative correlations (p≥0.05) between *Dialister*, *Dorea*, *Eggerthella*, and *Oscillospira* with GABA and choline. *Oscillospira* and *Eggerthella* were negatively correlated (p≤0.05) with GABA and choline, whereas *Eubacterium* and Lachnospiraceae were negatively correlated (p≤0.05) only with choline. At the species level, positive correlations (p≤0.05) were found for *C*. *difficile*, *D*. *formicigerans*, *E*. *lenta*, *F*. *prausnitzii*, Lachnospiraceae, *L*. *zeae*, *Oscillospira*, and *P*. *granolosum* with GABA, choline, glycine, and uracil. For OTUs and OTUs at the genus level, we found positive correlations (p≤0.05) for *Egghertella* with *Bulleidia*, *Dialister*, *Dorea*, *Eubacterium*, and Lachnospiraceae. A negative correlation was detected between *Egghertella* and Clostridiaceae (p≤0.05). At the species level, *C*. *difficile* was positively correlated with *Coprobacillus* (p≤0.05) but negatively correlated with *D*. *invisus*, *D*. *formicigerans*, *E*. *lenta*, *F*. *prausnitzii*, and *Ruminococcus* (p≤0.05). *D*. *invisus*, *D*. *formicigerans*, *E*. *lenta*, *F*. *prausnitzii*, and *Ruminococcus* were positively correlated (p≤0.05) (**S10 Table**).

## Discussion

This study illustrates an original fused metagenomics and metabolomics microbiota profile, characteristic of CF children luminal gut microbiota. This profiling revealed a CF meta-enterophenotype induced by microbial community plasticity and microbial metabolism alterations related to the physiopathology of the host mucosa. Considering the high variability of the CF phenotype, the present study focused on infants and preschool children. Infectious pulmonary exacerbation was an exclusion criterion. The metataxonomy hierarchical clustering showed that CF bacterial community levels were age-independent, contrary to the pattern found in HC subjects, in which the OTUs distribution was age-dependent, as expected [13]. Moreover, consistent with clinical stability, the lung microbiota of this cohort primarily was composed of *S*. *aureus* and *H*. *influenzae*, with only 5 patients manifesting *P*. *aeruginosa* infection. This is in agreement with the Annual Data Report of the CF Foundation (https://www.cff.org/Our-Research/CF-Patient-Registry/2015-Patient-Registry-Annual-Data-Report.pdf).

Gut microbiota OTUs patterns in CF children mostly overlapped that of younger HC and showed a low ecological complexity, suggesting an immature, early life-like ecological organization, which remained almost age-independent amongst children in the 1–6 year age range.

CF patients with lung *P*. *aeruginosa* colonization (LM3) showed a microbiota richness more similar to healthy subjects than to the other lung microbiota groups.

Moreover, even if the information derived from hypopharyngeal secrection, detected by cultured-dependent methods, were limited to investigate the wide complexity of microbial lung ecosystem, they were still able to provide a description of the system.

However, culture-independent methods may better highlight the lower airways of pediatric CF lung ecosystems which are characterized by complicated microbial environments which mutate with age and are influenced by several factors, including disease process, drug therapy, feeding habits and nutrition [11]. Hence, as reported by Hahn et al. [14], in order to deeply describe the entire lung microbial ecosystem and to put it in relation with the gut microbiota it would be necessary the application of metagenomic analysis by using next generation sequencing platforms which may generate results on microbial composition and structure [11,14].

Remarkably, an antibiotic therapy regimen did not significally impact the alpha-diversity of gut microbiota. Azithromycin plus antibiotic therapy for aerosol (A+AA) worsened alpha-diversity of NA, AT patients, compared to HC, while AA slightly increased the diversity, complementing the clinical debate on the actual effect of AA alone or A+AA on lung microbiota health [15]. Hence, to this purpose, Cuthbertson et al. [16] emphasizes the resilience as “the rate at which microbial composition returns to its original composition after being disturbed” regardless of the microbial district studied [17]. In particular, authors found that the microbiota did not significantly change in composition, indicating resistance to perturbation inside the lung after antibiotic treatment [16]. In our case, such resilience could also describe the CF gut microbiota profile, regardless antibiotic regimens.

Moreover, patients with azithromycin plus antibiotic therapy for aerosol could worsen the unbalanced gut condition of CF subjects. CF subjects receiving only inhaled therapy, show an “intermediate” diversity profile, more similar to HC, indicating thatantibiotic therapy for aerosol, by selectively modulating *P*. *aeruginosa*–linked lung microbiota, has a slight impact on gut microbiota diversity. Comparison between OTU abundance in CF and HC subjects, filtered by FDR, revealed higher abundance of *P*. *acnes*, *Staphylococcus* spp., and Clostridiaceae in CF compared to healthy subjects. In particular, *C*. *difficile* was only found in CF patients. However, *Eubacterium*, *D*. *formicigenerans*, and *E*. *lenta* were higher in healthy subjects, regardless of antibiotic therapy regimen or pancreatic function. The signicant abundance of *D*. *formicigerans*, *Eubacterium*, *E*. *lenta*, and Lachnospiraceae, combined with the sole presence of *F*. *prausnitzii* in HC, suggest conditions of microbiota “wellness”. Gut microbiota dysbiosis and the growth of *C*. *difficile* in CF could be facilitated by repeated courses of oral or inhaled antibiotics; however, our results show that the high abundance of *C*. *difficile* in CF seems to be associated with the disease. *C*. *difficile* infections have been associated with *Blautia*, *Pseudobutyrivibrio*, *Roseburia*, *Faecalibacterium*, *Anaerostipes*, *Subdoligranulum*, *Ruminococcus*, *Streptococcus*, *Dorea*, and *Coprococcus* depletion [18], similar to what we found in the present CF cohort. In CF patients affected by secondary bile acid alteration, a reduction in *Lachnospiraceae* and *Ruminococcaceae* was also found [19].

The fecal CF metabolic profile is characterized by high levels of alcohols (*i*.*e*., ethanol, 1-propanol) and esters (*i*.*e*., propylbutyrate, ethylacetate), suggesting a microbial strategy specializing in the removal or detoxification of acids or alcohols [20] and characterized by “unbalanced” intestinal microbiota. In this context, the ipercaloric diet followed by CF patients, could stimulate the growth of species such as *P*. *acnes* [10,19] and the depletion of genera such as *Roseburia* [21]. In a gut environment in which alcohol production is stimulated, *C*. *difficile* spore germination, growth, toxin production, and outgrowth of alcohols are facilitated [22].

In our cohort, integration of OTUs and metabolite data generated a fused predictive model which correctly classifies 85.4 ± 3.4% of samples which are characterized by high abundance of *Propionibacterium* and Clostridiaceae and low abundance of *Eggerthella*, *Eubacterium*, *Ruminococcus*, *Dorea*, and Lachnospiraceae in combination with high levels of GABA, choline, ethanol, propylbutyrate, and pyridine and low levels of sarcosine, 4-methylphenol, uracil, glucose, acetate, phenol, benzaldehyde, and methylacetate.

Loss of CFTR function leads to a relatively dehydrated luminal environment and a reduced pancreatic bicarbonate secretion with a consequent accumulation of mucus in the CF intestine. A similar pattern is shared by patients with compensated pancreatic functionality [23], supporting the reduced effect of bicarbonate secretion at gut level Altered ion transport induces, at the gut luminal level, the creation of a reducing acidic environment, promoting the selection and growth of anaerobic bacterial species such as *C*. *difficile* and *Propionibacterium* which, in turn, produce alcohols, esters, and pyridine in the absence of oxygen. In the same environment, species like *E*. *lenta* and *F*. *prausnitzii* as well as aldehydes, acids, carbohydrates, and phenols tend to decrease.

CFTR mutations inhibit the passage of Cl^-^ ions and water in the apical membrane of intestinal epithelial cells. Therefore, GABA production, becomes a compensatory mechanism [24] of lumen hydration overcoming poor electrolyte transport. GABA’s effects on the GI tract depend on the activation of ionotropic GABA_A_ and GABA_C_ and of metabotropic GABA_B_ receptors, regulating both excitatory and inhibitory signalling in the enteric neuronal system [25]. The biosynthesis of GABA occurs through the decarboxylation of glutamate by glutamate decarboxylase (GAD)[24]. Therefore, increased activity of GAD appears to provide positive feedback on GABA production, which induces Cl^-^ flow, replacing dysfunction of the *CFTR* gene. Hence, the ionotropic GABA receptors are Cl^-^ channels, which contribute to intestinal fluids and electrolyte transport modulation.

The results also pointing to high fecal choline levels in CF patients, which are in line with the hypothesis that impaired choline transport through the intestinal epithelium is occurring secondary to CFTR impairment [26]. Choline deprivation and altered hepatic phosphatidylcholine (PC) metabolism may affect plasma PC homeostasis and extrahepatic organ function in CF [19]. In fact, choline represents an essential nutrient for cell membrane PC formation, especially during proliferation and epithelial repair, as well as for the synthesis of very low density lipoproteins in the liver [27]. Therefore, changes in epithelial uptake of choline could have important consequences for the growth and wellness of CF patients.

**Fig 4:**
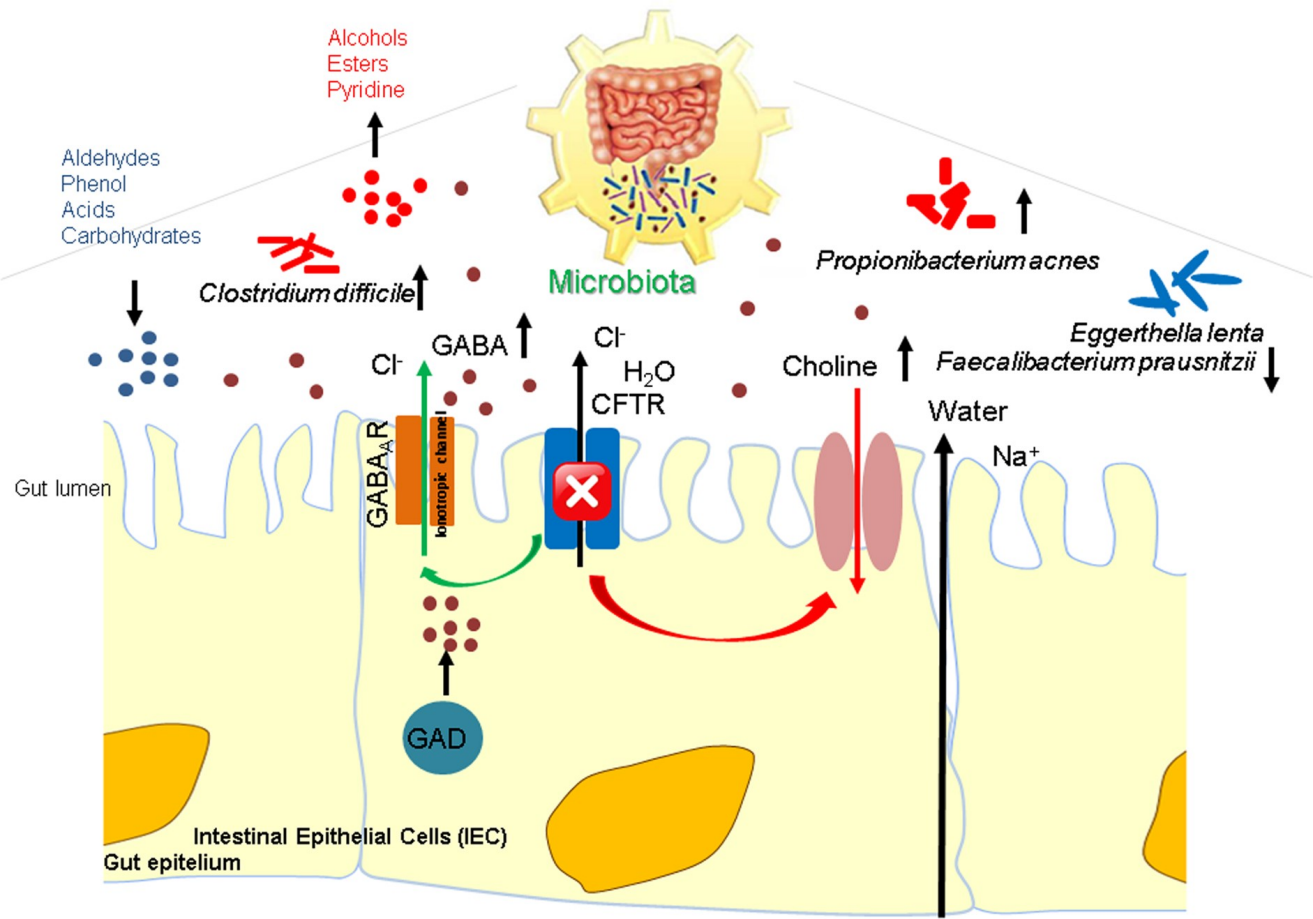
Functional descriptive model of CF gut microbiota predicting the host-microbiota interaction driven by CFTR impairment. Working model of the ionotropic GABA receptor, a ligand-gated Cl^-^ channel, and cystic fibrosis transmembrane conductance regulator (CFTR), another known Cl^-^ channel intestinal epithelial cells, and the related biochemical reaction in the presence of a CFTR deficiency. The CFTR system mutation lead to the block of Cl^-^ ions and water flow passage. GABA synthesized, stored, and secreted by small intestinal epithelial cells via GAD activity binds its type A receptors (GABA_A_Rs) in the apical membrane. GABA_A_Rs are opened, which causes Cl^-^ efflux to the intestinal lumen increasing fluid formation. In the gut microbiota, the new equilibrium produces a low pH and anaerobic envroment which stimulates the growth of microbial species as *Clostridum difficile*/*Propionibacterium* and a decrease in species such as *Eggerthella lenta*/*Faecalibacterium prausnitzii* which produce specific microbial metabolites (*i*.*e*., alcohols, esters, pyridine, aldehydes, and acids).

We envisage a functional “host-gut microbiota” multi-omics-based model in which microbial and metabolite variations are interrelated with CFTR functional defects (**Fig 4**).

Consistent with this model, CF-induced changes in the gut microbiota community and metabolome result in a distinct dysbiosis pattern secondary to impaired *CFTR* gene function, partially influenced by oral antibiotic therapy, which may result in the promotion of specific microbial anaerobic species. CF biomarkers, such as GABA and choline, may directly reflect alterations in the intestinal transport of water and cationic osmolytes resulting from CFTR dysfunction, while alcohols, esters, and pyridine a microbial imbalanced activity.

All together, these results suggest that the gut microbiota in children with CF is intrinsically linked to CFTR impairment, regardless of age, and with dysbiosis uninduced by pancreatic function, diet and in part related to oral antibiotic therapy or lung colonization/infection. Early interventions to enhance the “microbiota wellness” [28] of CF children may reduce the CF disease phenotype. Species like *F*. *prausntzii* and *E*. *lenta* might represent healthy biomarkers and potential probiotics in these patients.

## Methods

### Patient characteristics

A cohort of 31 consecutive CF patients aged 1 to 6 years (average age 3.7 years, SD ± 1.72; 13 males and 18 females) were recruited at the Cystic Fibrosis Unit of the Bambino Gesù Children’s Hospital (OPBG, Rome, Italy). The study protocol was approved by the OPBG Ethics Research Committee with signed informed consents.

The following phenomics data were collected from 31 patients with CF: age, gender, results of sweat chloride test, genotype (http://www.genet.sickkids.on.ca/app; http://www.cftr2.org), pancreatic function, meconium ileus status, BMI (>2 years of age) or Z-score (W/L of <2 years of age), lung colonization [29], history of antibiotics/probiotics usage, and complete phenotype expression (**S1 Table**). Diagnosis of CF was made based on results of a pathological sweat test (chloride >60 mmol/L, reference value), as described by Gibson and Cooke [30], or by the presence of 2 CF-causing mutations in the *CFTR* gene [31]. Patients were classified as follows: a) *complete disease espression*, indicating the presence of *CFTR* I, II, and III mutation classes and pancreatic insufficiency (PI); or b) *not complete disease espression*, indicating the presence of *CFTR* IV and VI mutation classes with pancreatic sufficiency (PS) (**S1 Table**; https://www.cftr2.org/).

Patients were age-matched (1:1) with a cohort of HC who were screened by means of a survey of the OPBG Human Microbiome Unit that involved gut microbiota programming. HC inclusion criteria were: absence of gastrointestinal infections and no antibiotic and pre-probiotic intake in the previous two months the sample collection.

Patients with CF were recruited under conditions of clinical stability (*i*.*e*., absence of pulmonary symptoms of infectious exacerbation). They received as chronic regimen a specific (bacteria-dependent) antibiotic pharmacotherapy for control of respiratory tract infection/colonization, including azithromycin, tobramycin (AA), and azithromycin plus tobramycin (A+AA) [30,31]. Amongst the 31 CF patients, 17 experienced pulmonary symptoms, hence an exacerbation regimen was practised. The exacerbation regimen was additive to chronic regimen for 10/17 and alternative for 7/17 patients. The exacerbation regimen was undertaken by administering a *per os* amoxicillin, quinolones and cephalosporins (**S3 Table**) [30,31]. Once symptons disapperead, patients had not been treated for acute infection at least for ≥2 weeks before collection of fecal specimens for gut microbiota analyses, but not longer to avoid clinical complications (**S3 Table**).

To assess the possible effect of both azithromycin and antibiotic for aerosol therapy on lung microbiota, a patient–therapy substratification was performed. Antibiotic chronic therapy were subcategorized as follows: i) NA, for patients not receiving antibiotic treatment (17/31); ii) AA, for patients receiving antibiotic therapy only for aerosol (7/31); iii) A+AA, for those undergoing in therapy with azithromycin plus antibiotic for aerosol (7/31) (**S1** and **S3 Tables**).

Consumption of the probiotic *Lactobacillus casei* strain GG, in the 3 months previous to the study, was suspended 15 days before fecal sampling. In terms of nutritional status, all patients followed a high-energy diet of 120% to 150% of the recommended dietary allowance (RDA) [32,33]. Nutritional status was evaluated by using the Z-score index for patients younger than 2 years and by using the BMI percentile for patients older than 2 years. Fecal elastase levels were used to evaluate PI (≤100 μg/g feces). Neither symptoms nor diagnoses of gastrointestinal diseases were registered at stages of gut microbiota analyses.

Only age, gender, and Z-score (W/L) or BMI were recorded for HC; these metrics were configured as discrete values (**S2 Table**).

The study protocols (534/RA; 1113_OPBG_2016) were approved by the OPBG Ethics Research Committee, in accordance with the Declaration of Helsinki (as revised in Seoul, Korea, October 2008). All parents/legal guardians gave written informed consent.

All fecal samples collected from HC and patients with CF were handled at the OPBG Human Microbiome Unit for biobanking and meta-omics processing. After sample collection, 3 of 31 CF samples were excluded for insufficient sample collection. For investigation of gut microbiota, metagenomics and metabolomics analyses were performed.

### Metagenomic analysis

#### DNA extraction

Genomic DNA was extracted from the 59 (28 CF and 31 HC) successfully collected fecal samples. Stools (200 mg) were resuspended in 1.5 mL of phosphate-buffered saline (PBS), homogenized by vortexing for 2 minutes, and centrifuged at 20,800*g*. After removal of the supernatant, the pellet was suspended in 500 μL of PBS, added as 500 μL of beads/PBS (1 mg/μL, w/v) (glass beads, acid-washed; Sigma-Aldrich, Milan, Italy). The 1:1 mixture was homogenized by vortexing for 2 minutes and was centrifuged at 5200*g* for 1 minute. The supernatant was collected and treated for 1 freeze–thaw cycle (-20°C/70°C) for 20 minutes per step. After centrifugation at 5200*g* for 5 minutes, the supernatant was processed by extraction with a QIAamp DNA Stool Mini Kit (Qiagen, Germany), according to the manufacturer’s instructions. DNA was eluted into 50 μL of purified H_2_O (Genedia, Italy). DNA yield was quantified using a NanoDrop ND-1000 spectrophotometer (NanoDrop Technologies, Wilmington, DE). The concentration of DNA was adjusted to 10 ng/μL and was applied as a template for 16S rRNA metagenomic 454 sequencing analyses.

#### 16S rRNA–targeted metagenomic 454 sequencing

The gut microbiome was probed by pyrosequencing the V1 to V3 regions of the 16S rRNA gene (amplicon size, 520 base pairs [bp]), on a GS Junior platform (454 Life Sciences, Roche Diagnostics, Italy), according to the pipeline described by De Filippis et al. [34].

Bioinformatics

Raw reads were filtered by means of 454 amplicon signal processing; sequences were analyzed with Quantitative Insights into Microbial Ecology (QIIME, version 1.7.0) software [35]. To ensure high accuracy of detection of OTUs, we excluded from analysis all post-demultiplexing reads of <300 bp in length, with an average quality score <25, and with ambiguous base calling.

Sequences that passed the quality filter were denoised [36], and singletons were excluded. OTUs defined by a 97% similarity were selected *de novo* using the uclust pipeline [37]. Representative sequences were submitted to the RDPII classifier [38] to obtain the taxonomy assignment; the relative abundance of each OTU was determined by means of the Greengenes 16S rRNA gene database [39].

Ecological diversity for each sample was described in terms of the following: i) the number of OTUs obtained for each sample; ii) the Chao1 metric; iii) the Shannon index; and iv) the estimated sample (number) coverage (ESC). The beta-diversity—which represents a comparison of microbial communities based on their dissimilar compositions—was computed with the unweighted UNIFRAC algorithm and was imported in R to obtain plots of principal coordinates analysis (PCoA).

The alpha and beta-diversity assessment and the Kruskal Wallis test were conducted by means of QIIME software, using “alpha_rarefaction.py, beta_diversity_through_py, group_significance.py” scripts, as previously described [34]. Correction of p values for multiple testing was performed when necessary [40]. A univariate statistical analysis of OTUs was performed by the Mann-Whitney U Test using IBM SPSS statistical software, version 21.

The correlation between abundance of OTUs and subjects was computed using a heat plot drawn in R (function *heatplot* of the made4 package, Bioconductor v2.5) with hierarchical ward-linkage clustering based on Spearman’s correlation coefficient of the level of microbial genera. Only genera present in ≥10% of samples were included. Average values of Chao1 index, derived from α diversity, were compared for the following data pairs: i) patients with CF vs. HC (Student’s *t* test); ii) pancreatic function vs. HC; and iii) antibiotic therapy vs. HC (Tukey's range test). A quantitative comparison of OTUs distributions was made with the Kruskal-Wallis test for CF disease, pancreatic function, and antibiotic therapy variables vs. HC reference values. Statistical significance was given as the output value (QIIME). Normality of distribution was ascertained for all data using the Shapiro-Wilk normality test.

For discrete variables (*e*.*g*., sex, presence of disease), permutational multivariate analysis of variance using distance matrices (ADONIS) and analysis of similarities (ANOSIM) were run, using the R package vegan (CRAN, Community Ecology Package), to verify the influence of these variables on the microbial population.

Availability of data

The following 16S rRNA sequences were deposited in the sequence-read archive (SRA) of NCBI (https://www.ncbi.nlm.nih.gov/; submission IDs): SUB439815, SUB682236, SUB688648, SUB688650, SUB693641, SUB693678, SUB701127, SUB701163, SUB703465, SUB705046, SUB705055, SUB707306, SUB707310, SUB708551, SUB708584, SUB713156, SUB742829, SUB743408, SUB744094, SUB745394, SUB745451, SUB747212, SUB747218, SUB748238, SUB748244, SUB748368, SUB748388, SUB749246, SUB750378, SUB750382, SUB751581, SUB751693, SUB754325, SUB755571, SUB755577, SUB755743, SUB756623, SUB756702, SUB757375, SUB757613, SUB759548, SUB759553, SUB764480, SUB768026, SUB769359, SUB770031, SUB770058, SUB770094, SUB770126, SUB770134, SUB770126, SUB770470, SUB770508, SUB771512, SUB771523, SUB771543, SUB771548, SUB771555, SUB772418.

### Metabolomic analysis

#### Generation of volatilome by GC-MS/SPME

Following preconditioning, the carboxen-polydimethylsiloxane–coated fiber (CAR-PDMS) (85 μm) and the manual SPME holder (Supelco Inc., Bellefonte, PA) were used for extraction of VOCs, according to the manufacturer’s instructions. Before exposure to the headspace, the fiber was exposed to the GC inlet at 250°C for 5 minutes for thermal desorption. An average quantity of 100 to 500 mg of feces recovered from each sample (*i*.*e*., 28 CF and 31 HC fecal samples, respectively) was considered suitable for processing. These specimens were placed into 10-mL glass vials and were combined with 4-methyl-2-pentanol (final concentration 400 mg/ml) as an internal standard. Fecal samples were then equilibrated at 45°C for 10 minutes. The SPME fiber was exposed to each sample for 45 minutes. Phases of equilibration and absorption were carried out with constant stirring. The fiber was inserted into the GC injection port (10 minutes) for sample desorption, as previously described [41]. GC-MS analyses then were conducted on a Hewlett-Packard 6890 GC coupled to a 5973C mass-selective detector operating in electron-impact mode (ionization voltage, 70 eV); this was housed within a system fitted with a 1-mm quartz liner equipped with a Supelcowax 10 capillary column (length, 60 m; inner diameter [ID], 0.32 mm; Supelco, Bellefonte, PA). The following thermal program was applied: 50°C for 1 minute, a temperature increase by 4.5°C per minute to 65°C and then by 10°C per minute to 230°C, and a hold at 230°C for 15 minutes. Injector, interface, and ion-source temperatures were 250°C, 250°C, and 260°C, respectively. The total run time was 35.83 minutes. The mass-scan range was 30 to 300 a.m.u. at 5.19 scans per second. Injections were carried out in splitless mode, under helium carrier (1.5 mL/min). Molecules were identified on the basis of their retention times (RTS), relative to pure compound RTS (Sigma-Aldrich, Milan, Italy). Chromatograms were integrated and identified by comparing the fragment pattern with those in the mass spectral NIST library (version 2d, built April 26, 2005; National Institute of Standards and Technology, Rockville, MD) and literature [42]. This was followed by manual visual inspection. Quantitative compound data were obtained by interpolation of the relative areas versus the area of the internal standard. Data were expressed in ppm (mg/kg).

#### Generation of non volatile compounds by ^1^H-NMR

In order to performe the ^1^H-NMR analysis an average quantity of 100 to 500 mg of feces recovered from each sample was combined with 1 mL of D_2_O–PBS buffer. Each sample was vortexed for 2 minutes and then centrifuged for 40 minutes at 4000*g* and 20°C to obtain fecal water. In total, 600 μL of supernatant was collected and analyzed.

In our analysis of fecal-water specimens, the *J*-resolved pulse sequence was used to better observe resonances, which were partially or completely buried in a typical 1-dimensional (1D) spectrum. This sequence allows for an enhanced quality of extracted metabolic information. Two-dimensional (2D) ^1^H *J*-resolved (JRES) NMR spectra were acquired on a Varian Unity Inova 500 MHz spectrometer with a double-spin echo sequence. Sixteen scans were performed per increment for a total of 32 increments, and 16 K data points were collected. The spectral widths were 6 kHz in F2 and 40 Hz in F1, and the relaxation delay was 3.0 seconds. The water resonance was suppressed with presaturation. Each free induction decay (FID) was multiplied with sine-bell window functions in both dimensions and Fourier transformed. JRES spectra then were tilted by 45°, made symmetric about F1, and referenced to acetate at delta_H_ = 1.90 ppm. Proton-decoupled skyline projections (p-JRES) were exported using VnmrJ 3.2 software [43]. Metabolites were identified based on data in previous reports, and the assignment was confirmed by 2D homo- and heteronuclear NMR spectroscopy experiments. Data were expressed in arbitrary units.

#### ^1^H-NMR spectra pre-processing treatment

Exported 1D skyline projections were aligned and then reduced into spectral bins with widths ranging from 0.01 to 0.03 ppm by the advanced chemistry development (ACD) intelligent bucketing method (1D NMR Manager software; ACD Labs, Toronto, Canada). Binning was performed with the residual water region excluded (delta, 4.66–4.80 ppm). The resulting bins were integrated and normalized with respect to the total integral region to generate the data matrix for multivariate analysis.

### Microbiological analyses

#### Culturomics of hypopharyngeal secretions

Lung microbiota culturomics (CU)-based analyses were included to microbiologically monitor and characterize respiratory tract infections and colonizations on hypopharyngeal secretions, collected by aspiration, (**S1 Table**) since the patients were unable to expectorate according to age. The lung colonization/infection pattern was prevalently monomicrobial, in part because the cohort comprised young subjects (1–6 years of age) and only main pathogens (*i*.*e*., core pathogens) were detected by CU. All specimens were collected in compliance with local guidelines approved by the OPBG Health Directorate and were processed in accordance with standard microbiological guidelines [44]. For aerobic and facultative anaerobic bacterial cultures, serial 10-fold dilutions of each hypopharyngeal secretion (HS) specimen in brain infusion broth (BHI) were carried out at room temperature (RT). Subsequently, 10 μL of each serial 10-fold dilution, up to 10^−5^, were streaked on plates with the following solid media: Columbia blood agar, with 5% sheep blood (COS); mannitol-salt agar (MSA); MacConkey agar (MAC); cetrimide-agar; chocolate agar, with or without bacitracin (CHA+BAC; CHA); and Colistin-nalidixic acid blood agar (CNA). Plates were incubated aerobically at 35°C ± 2°C -with the exception of CNA and chocolate agar cultures, which were incubated in the presence of 5% CO_2_- for 2 days. Representative single colonies of aerobic/microaerophilic Gram-positive bacteria (*e*.*g*., *S*. *aureus*, *S*. *pneumoniae*, other Streptococcaceae), Gram-negative bacteria (*e*.*g*., *Pseudomonas* spp., *Burkholderia* spp., other glucose nonfermenting Gram-negative bacilli), and fastidious bacteria presumptively were identified based on colony morphology, growth at different temperatures or on selective media, Gram reaction, oxidase-catalase activity, motility, and pigment production. Species identification then was made in a CU matrix-assisted laser desorption/ionization time of flight mass spectrometry (MALDI-TOF-MS) based approach with a MALDI-TOF MS Biotyper (Bruker Daltonics, Germany) [45]. Aerobic bacterial isolates, for which conventional phenotypic identification was not univocal (*Burkholderia cepacia* complex; other *Burkholderia-*like bacteria, such as *Burkholderia gladioli*, *Ralstonia* spp., *Pandorea* spp., and *Inquilinus limosus*), were identified by means of amplification and sequencing of 16S rRNA (MicroSEQ 500 16S rDNA sequencing kit, ThermoFisher), sequencing of *recA* genes, and species-specific PCR [46,47]. To classify CU-based lung microbiota groups, the following criteria were applied: absence of microbial colonizers or pathogens and presence of *H*. *influenzae*, *S*. *pneumoniae*, *E*. *coli*, *E*. *coli* ESBL+, *Serratia marcescens*, *E*. *cloacae*, and *B*. *catarrhalis* was deemed group 1; presence of methicillin-resistant *S*. *aureus* (MRSA), *S*. *malthophilia*, *E*. *corrodens*, *Acinetobacter* spp., and *F*. *meningosepticum* was deemed group 2; presence of *P*. *aeruginosa*, and *Pseudomonas* spp. was deemed group 3. Based on these criteria, 10 patients were assigned to LM 1, 14 to LM 2, and 4 to LM 3.

### Statistical analysis

#### Data classification, generation of fused data, and model validation

To characterize the differences between CF and HC subjects from a multi-omic standpoint (*i*.*e*., ^1^H-NMR- and GC-MS–based metabolomics and metagenomics), chemometric classification methods were adopted. The aim of these classification techniques is to build mathematical models that can discriminate among 2 or more classes (*i*.*e*., groups of individuals sharing similar characteristics). In the present study, the 2 classes were the group of HC and the group of CF patients. For reduction of multidimensional data, the raw data matrix of OTUs was condensed to a matrix of OTUs measured in ≥80% of specimens in CF and HC groups. The reduced genus matrix included the following 31 of 165 variables: 1) *Actinomyces*; 2) Bacteroides; 3) *Bifidobacterium*; 4) *Blautia*; 5) Clostridiaceae; 6) *Clostridium*; 7) *Collinsella*; 8) *Coprococcus*; 9) *Corynebacterium*; 10) *Eggerthella*; 11) *Enterobacter*; 12) Enterobacteriaceae; 13) *Enterococcus*; 14) Erysipelotrichaceae; 15) *Escherichia*; 16) *Eubacterium*; 17) *Faecalibacterium*; 18) *Dorea*; 19) *Dialister*; 20) *Gemella*; 21) *Granulicatella*; 22) Lachnospiraceae; 23) *Lactobacillus*; 24) *Propionibacterium*; 25) *Roseburia*; 26) Ruminococcaceae; 27) *Ruminococcus*; 28) *Streptococcus*; 29) Turicibacteraceae; 30) *Veillonella*; 31) others. For ^1^H-NMR and GC-MS procedures, raw data matrices were not reduced. The homonymous partial least-squares (PLS) regression tool has utility for classification of data sets characteristics (*i*.*e*., a high number of variables with statistically significant correlations) that had been processed with PLS discriminant analysis (*i*.*e*., PLS-DA) [48,49]. Any regression method can be turned into a classifier by introducing a dummy dependent variable **Y**, which carries the information for classes belonging to a 2-class/binary problem, as in the present study. **Y** is a binary vector with as many entries as the number of samples. Accordingly, if an individual is healthy, his or her corresponding y is 0. If the individual is ill, the corresponding y is 1. With these data, a regression model can be built between the matrix of predictors **X** (*i*.*e*., the instrumental data recorded on the samples; either GC-MS, ^1^H-NMR, or metagenomics outcomes) and the dummy-coded **Y**. For each observation, the model returns a predicted value of the response (*i*.*e*., of **Y**). This value is not binary and can be any real number. Sample classification is accomplished by placing a threshold on predicted **Y** values: in a 2-class problem, in which the first class is coded 0 and the second 1, typically the threshold value is 0.5. Therefore, if the predicted **Y** is below the threshold, the sample is assigned to the first class; if above the threshold, the sample is assigned to the second class.

In PLS-DA, the regression model is built using the PLS algorithm; this algorithm differs from multiple linear regression because it involves data matrices with many correlated variables. Hence, PLS-DA is useful for processing outcomes from instrumental -omics techniques. PLS operates by projecting the data matrices onto a low-dimensional subspace of orthogonal latent variables (LV) (*i*.*e*., abstract), characterized by the directions of higher covariance between **X** and **Y**. PLS then applies the coordinates of observations onto these new sets of variables (scores), as predictors onto which regresses the dependent variable. In a second stage of analysis (more than 1 block of experimental variables was available), the authors attempted to integrate information from the individual data matrices into a single model, by means of a low-level data fusion protocol. In this protocol, the individual data blocks (in the present case, the matrices from ^1^H-NMR, GC-MS, and targeted-metagenomics) were concatenated to form a single augmented data matrix **X**_**LL**_**: *X***_*LL =*_ [***X***_***NMR***_***X***_***GC-MS***_***X***_***meta***_]. This matrix was used for further modeling (*e*.*g*., for building a classification model by means of PLS-DA). Because the 3 blocks of data came from different experimental techniques and had different variances (to prevent any from prevailing on the others) and because of high inherent variability, the data were block-scaled prior to concatenation. That is, the data were divided by their Frobenius’ norm (regarded as the standard deviation of the whole block), to remove this difference. To ensure reliable interpretation of results with individual matrices and fused data—especially for the microbial biomarkers and identified metabolic pathways—the chemometric models were validated. The scope of the validation phase was to verify that the model had identified a significant class structure. In other words, results of validation demonstrated a real connection, rather than chance correlations, between the experimental data and class labels of the samples. As suggested by Szymańska et al. [50] for -omic studies, we carried out validation by means of a combination of double cross-validation (CV) and permutation tests.

Validation should not be conducted on the same samples used for model building. Because PLS-DA involves a model selection stage to select the optimal complexity (*i*.*e*., the number of LV), an additional optimization step is needed. Given that model optimization and the assessment of model quality need to be carried out independently, the use of double CV is crucial. Double CV consists of 2 nested loops, usually labeled CV1 (inner loop) and CV2 (outer loop). The aims of this assessment are the selection of the optimal number of LV_n_ and the evaluation of the predictive ability of the model. In the outer loop, the data were divided into a training set and a test set. The test set was applied to validate the model whose optimal complexity is evaluated on the training set using an inner CV loop. In CV1, the training set is split into calibration and optimization sets: the calibration set is utilized to build models with an increasing number of components (from 1 to LV_max_); these components then are applied to the optimization set. The complexity that yields the lowest error is then selected for the final model. This model is validated on the test set from CV2. It is known that even very good results can be obtained just by causal selection of the data splits. To assess statistical significance of obtained figures of merit (*i*.*e*., of model quality), permutation tests were performed, and the experimental nonparametric distribution of the null hypothesis and accompanying p value were obtained. Three diagnostic statistical tests were applied in the optimization and performance assessment of PLS-DA: 1) the NMC; 2) the AUROC; and 3) the DQ^2^. PLS-DA models obtained with NMC or AUROC are more powerful in detecting very small differences than are models obtained with DQ^2^. Therefore, NMC and AUROC have been recommended in 2-group discrimination metabolomics studies [50]. All analyses were carried out with in-house routine programs, written in MATLAB software (MATLAB R2013b, The Mathworks Inc, Natick, MA).

## Supporting information

S1 Fig Beta-diversity analysis. (DOC)

S2 Fig Bar chart representing the OTU relative abundances in HC and CF. (DOCX)

S3 Fig Graphical representation of Kruskal-Wallis test. (DOC)

S4 Fig PLS-DA results of GC-MS/SPME analysis. (DOC)

S5 Fig PLS-DA results based on ^1^H-NMR analysis. (DOC)

S1 Table Clinical features of CF patients: phenomic metadata. (DOC)

S2 Table Clinical features of HC subjects: Phenomics metadata. (DOCX)

S3 Table Antibiotic therapy under a chronic regimen. (DOC)

S4 Table Raw OTU data. Genus 80% reduced.
(XLS)

S5 Table Number of sequences analyzed, α-diversity indices (ChaoI and Shannon), and estimated sample coverage (ESC) for 16S rRNA amplification from DNA extracted from CF and HC fecal specimens. (DOC)

S6 Table Univariate analysis of OTUs, depicting significant differences by the Mann-Whitney U test. (DOC)

S7 Table GC-MS/SPME metabolites grouped by chemical class: Raw data, total, mean, p-values, FDR adjusted p-value, interquartile range (IQR), and distribution frequency of samples. (XLS)

S8 Table ^1^H-NMR metabolites grouped by chemical class: Raw data, total, mean, p-values, FDR adjusted p-value, interquartile range (IQR), and distribution frequency of samples. (XLS)

S9 Table Spearman’s correlation and p-values of VOCs and OTUs at phylum, family, genus and species levels. (XLS)

S10 Table Spearman’s correlation and p-values of ^1^H-NMR metabolites and OTUs at phylum, family, genus and species levels. (XLS)
