# Supplementary material for: Gut microbiota signatures in cystic fibrosis: Loss of host CFTR function drives the microbiota enterophenotype
Source: PLoS One. 2018 Dec 6;13(12):e0208171. doi: 10.1371/journal.pone.0208171 (PMC6283533; doi:10.1371/journal.pone.0208171)
Supplement: S1 Fig — (DOC) [file pone.0208171.s001.doc]

**S1 Fig.**


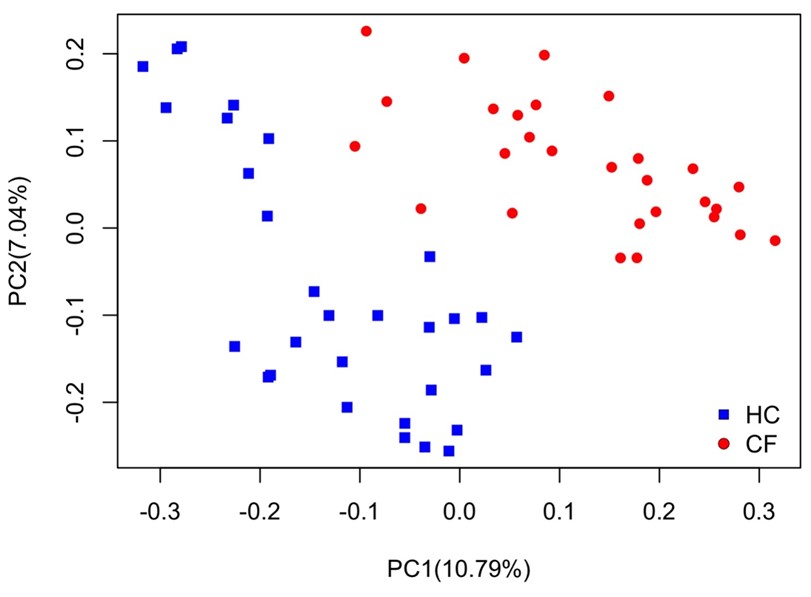
 **S1 Fig. Title. *Beta-diversity analysis***.

**Legend**. 2D principal coordinates plot of unweighted UniFrac distances for 16S rRNA gene sequence data from CF patients (blue dot) and HC (red squares).
