# Supplementary material for: Gut microbiota signatures in cystic fibrosis: Loss of host CFTR function drives the microbiota enterophenotype
Source: PLoS One. 2018 Dec 6;13(12):e0208171. doi: 10.1371/journal.pone.0208171 (PMC6283533; doi:10.1371/journal.pone.0208171)
Supplement: S2 Fig — (DOCX) [file pone.0208171.s002.docx]

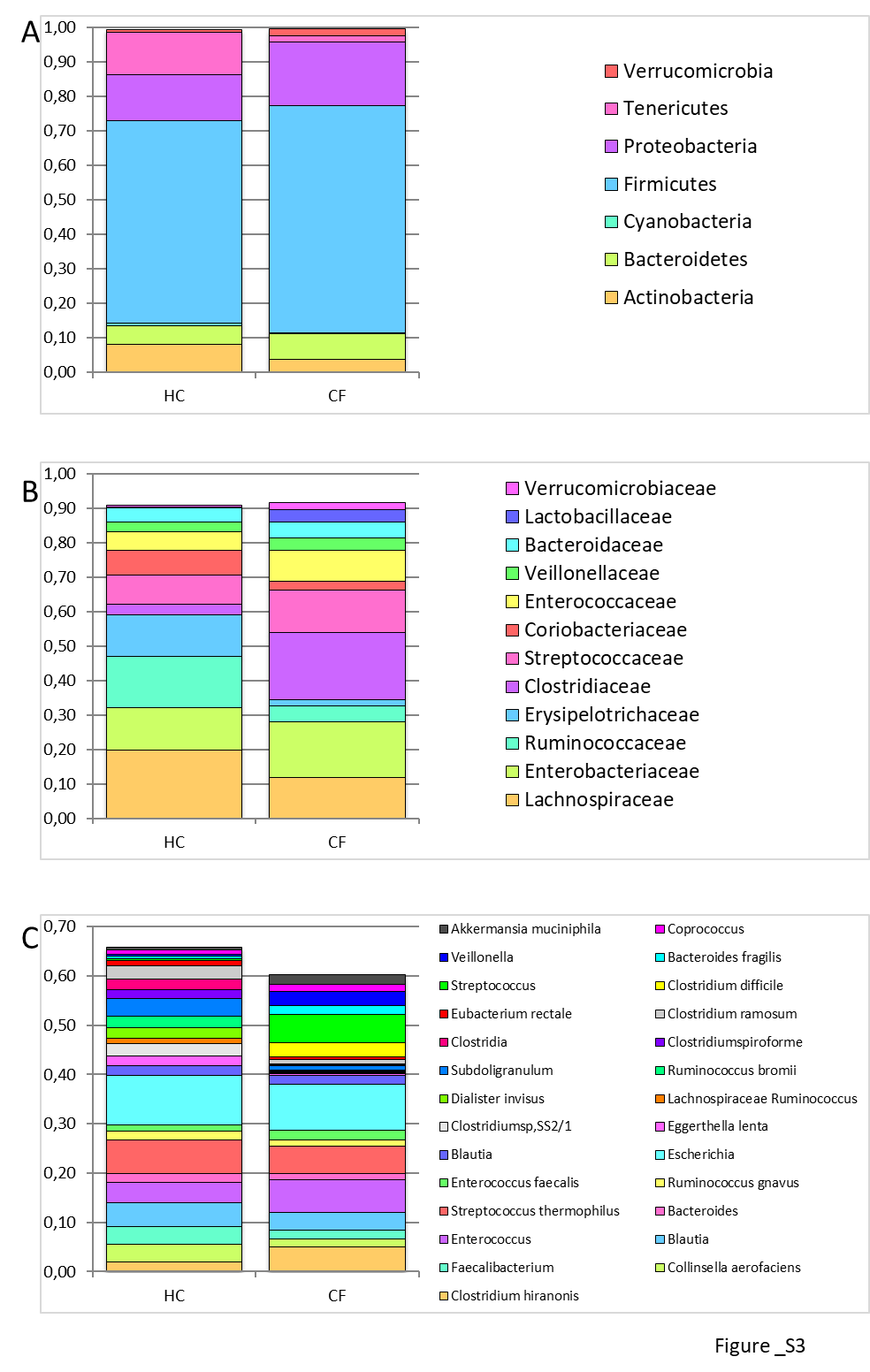


**S2 Fig.**

**Legend.** Bar chart representing the OTU relative abundances in HC and CF at phylum (**Panel A**), family (**Panel** **B**) and species (**Panel** **C**) levels. The OTUs reported in the bar charts have been selected for relative abundance >0.01.
