# Supplementary material for: Gut microbiota signatures in cystic fibrosis: Loss of host CFTR function drives the microbiota enterophenotype
Source: PLoS One. 2018 Dec 6;13(12):e0208171. doi: 10.1371/journal.pone.0208171 (PMC6283533; doi:10.1371/journal.pone.0208171)
Supplement: S3 Fig — (DOC) [file pone.0208171.s003.doc]

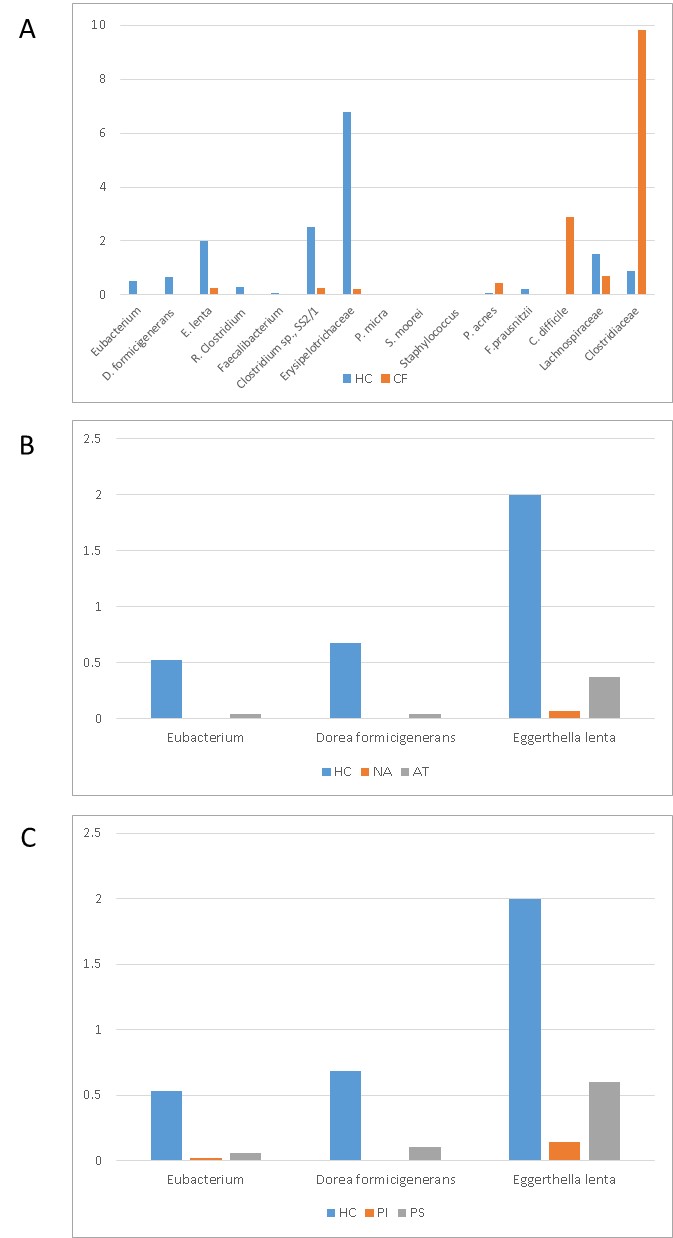


**S3 Fig.**

***Title. Graphical representation of Kruskal-Wallis test***

**Legend.** Bar chart reporting Kruskal-Wallis test results from Genus/Species distribution in: HC vs. CF (**Panel A**); HC vs. NA vs. AT (**Panel B**), HC vs. PI vs PS (**Panel C**). Only comparisons with a statistical significant p values were reported.
