# Supplementary material for: Gut microbiota signatures in cystic fibrosis: Loss of host CFTR function drives the microbiota enterophenotype
Source: PLoS One. 2018 Dec 6;13(12):e0208171. doi: 10.1371/journal.pone.0208171 (PMC6283533; doi:10.1371/journal.pone.0208171)
Supplement: S4 Fig — (DOC) [file pone.0208171.s004.doc]

**S4 Fig.**

**
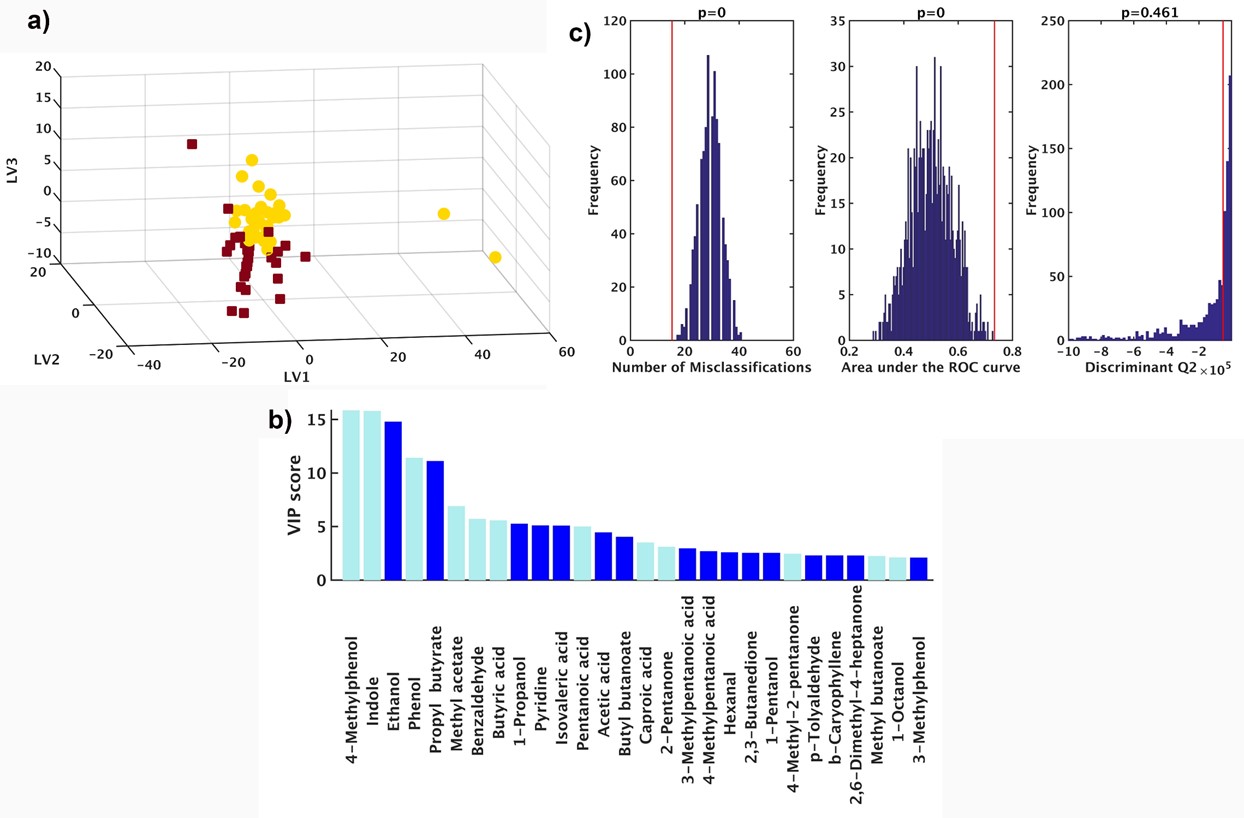
**

**S4 Fig.**

**Title.** ***PLS-DA results of GC-MS/SPME analysis***.

**Legend.** **Panel** **A.** Plot of LV scores: yellow, HC; dark red, CF patients. **Panel** **B.** Histograms representing VIP values: pale blue, low levels; dark blue, high levels (in CF patients). **Panel C.** Figures of merit relative to NMC, AUROC, and DQ^2^.
