# Supplementary material for: Gut microbiota signatures in cystic fibrosis: Loss of host CFTR function drives the microbiota enterophenotype
Source: PLoS One. 2018 Dec 6;13(12):e0208171. doi: 10.1371/journal.pone.0208171 (PMC6283533; doi:10.1371/journal.pone.0208171)
Supplement: S1 Table — (DOC) [file pone.0208171.s006.doc]

| **S1 Table.** Clinical features of CF patients: phenomic metadata | | | | | | | | | | | |
| --- | --- | --- | --- | --- | --- | --- | --- | --- | --- | --- | --- |
| **Patient code** | **Age**  **(years)** | **Gender** | **Sweat test**  **(chloride mmol/L)** | **Genotype** | **Pancreatic status** | **Meconium ileus** | **Z-score/**  **BMI^1^** | **Lung colonization/infection^2^** | **Use of antibiotics^4^** | **Use of probiotics** | **Complete phenotype expression** |
| P-06-2 | 6 | M | 77 | F508del/del2,3 | insufficient | yes | -1.5 | *H. influenzae, B. catharralis,*  *S. aureus*  (LM2)**^3^** | NA | no | yes |
| P-06-3 | 6 | F | 82 | F508del/F508del | insufficient | yes | -1.0 | *S. pneumoniae, H. influenzae* (LM1) | NA | no | yes |
| P-06-6 | 6 | M | 81 | F508del/L1065P | insufficient | no | 2.1 | Presence of viridans bacteria(LM1) | A+AA | no | yes |
| P-06-7 | 6 | F | 95 | F508del/F508del | insufficient | no | -1.0 | *H. influenzae*  (LM2) | AA | no | yes |
| P-06-8 | 6 | F | 125 | F508del/F508del | insufficient | no | 0.1 | *S. aureus, E.coli*  (LM2) | NA | no | yes |
| P-07-1 | 5 | M | 91 | F508del/F508del | insufficient | yes | -1.0 | *F. meningosepticum, Acinetobacter* spp.  (LM2) | A+AA | yes | yes |
| P-07-4 | 5 | F | 80 | 621+1G>T/R553X | insufficient | yes | -0.8 | *S. pneumoniae*  (LM1) | NA | no | yes |
| P-07-5 | 5 | M | 98 | F508del/G1244E | insufficient | no | 0.6 | Absent  (LM1) | NA | no | yes |
| P-07-6 | 5 | F | 121 | 621+1G>T/1898+G>A | insufficient | no | 2.5 | *S. aureus, H. influenzae,*  *B. catharralis*  (LM2) | AA | no | yes |
| P-07-7 | 5 | F | 94 | F508del/N1303K | insufficient | no | 0 | *S. aureus*  (LM2) | A+AA | no | yes |
| P-07-9 | 5 | F | 95 | F508del/M1V | insufficient | no | 0.3 | Presence of viridans bacteria(LM1) | NA | no | no |
| P-07-10 | 5 | F | 89 | F508del/P5L | sufficient | no | 1.0 | *B. catharralis*  (LM1) | NA | no | no |
| P-08-2 | 4 | M | 98 | F508del/R1162X | sufficient | no | 0.4 | *P. aeruginosa*  (LM3) | AA | no | yes |
| P-08-3 | 4 | M | 140 | F508del/712-1G>T | insufficient | no | 2.1 | Absent  (LM1) | NA | yes | yes |
| P-08-4 | 4 | F | 99 | F508del/S549A>C | insufficient | no | -0.1 | *P. aeruginosa, S. marcescens,*  *S. aureus, E. clocacae*  (LM3) | A+AA | no | yes |
| P-08-6 | 4 | M | 100 | F508del/F508del | insufficient | no | -1.0 | *S. aureus, E. coli* ESBL+,  *H. influenzae, E. corrodens* (LM2) | AA | no | yes |
| P-08-8 | 4 | F | 95 | F508del/R553X | insufficient | no | -0.2 | MRSA*, H. influenzae*  (LM2) | NA | yes | yes |
| **Patient code** | **Age**  **(years)** | **Gender** | **Sweat test**  **(chloride mmol/L)** | **Genotype** | **Pancreatic status** | **Meconium ileus** | **Z-score/**  **BMI^1^** | **Lung colonization/infection^2^** | **Use of antibiotics^4^** | **Use of probiotics** | **Complete phenotype expression** |
| P-09-2 | 4 | F | 77 | N1303K/5T-12TG | sufficient | no | 0.44 | *S. aureus, S. pneumoniae*,  *H. influenzae, A. xylosoxidans* (LM2) | NA | no | no |
| P-09-4 | 3 | M | 93 | N1303K/2184delAA | insufficient | yes | -1.27 | *K. oxytoca, S. marcescens,*  *S. maltophilia*  (LM2) | A+AA | no | yes |
| P-09-6 | 3 | M | 96 | F508del/F508del | insufficient | yes | -0.48 | *E. coli* ESBL+, *A. lowoffii,*  *C. parasilosis*  (LM1) | A+AA | yes | yes |
| P-09-7 | 3 | M | 90 | F508del/R1070Q | insufficient | no | 3.2 | *P. aeruginosa*  (LM3) | AA | no | yes |
| P-10-2 | 3 | F | 78 | F508del/S1253R | insufficient | yes | -0.4 | *S. aureus*  (LM2) | AA | yes | yes |
| P-10-4 | 2 | F | 85 | F508del/G542X | insufficient | no | -0.1 | *E. coli* ESBL+  (LM1) | A+AA | no | yes |
| P-10-7 | 2 | M | 63 | 1717-1G>A/D1152H | sufficient | no | -0.7 | *P. aeruginosa, S. aureus*  *H. influenzae*  (LM3) | AA | no | no |
| P-10-13 | 2 | M | 81 | 1717-1G>A/S1455X | sufficient | no | 0.6 | Presence of viridans bacteria  (LM1) | NA | no | no |
| P-11-3 | 2 | F | 61 | G542X/N187K | sufficient | no | 0.54 | *P. aeruginosa, S. maltophilia,*  *E. cloacae*  (LM3) | NA | no | no |
| P-11-4 | 1 | M | 114 | F508del/W1282X | insufficient | no | 1.3 | *S. aureus, E. cloacae, E. coli* (LM2) | NA | no | yes |
| P-11-7 | 1 | F | 100 | 1717-1G>A/Unknown | insufficient | no | -1.2 | *S. aureus*  (LM2) | NA | no | yes |
| P-11-8 | 1 | F | 84 | F508del/3659delC | insufficient | yes | -3.1 | *S. aureus*  (LM2) | NA | no | no |
| P-11-9 | 1 | F | 82 | F508del/E831X | sufficient | no | -0.3 | MRSA*, E. coli, H. influenzae* (LM2) | NA | no | yes |
| P-11-10 | 1 | F | 95 | F508del/1717-1G>A | insufficient | yes | 1.0 | *E. coli* ESBL+  (LM1) | NA | no | yes |

^1^Z-score: W/L for patients under 2 years of age, BMI: for patients older than 2 years of age.

^2^ESBL+: extended spectrum beta-lactamase-positive; MRSA: methicillin-resistant *Staphylococcus aureus.*

^3^Lung microbiome (LM) group: LM1: Presence of viridans bacteria *H. influenzae*, *S. pneumoniae*, *E.coli*, *E.coli* ESBL+, *S. marcescens*, *E. cloacae*, *B. catharralis*; LM2: *S. aureus*, MSSA and MRSA*, S. maltophilia*, *E. corrodens*, *Acinetobacter* spp., *A. xylosoxidans*; *F. meningosepticum, C. parasilosis, K. oxytoca*, *A. lwoffii;* LM3: *P. aeruginosa*

^4^Referred to antibiotic chronic regimen; NA, no antibiotic therapy; AA, antibiotic therapy for aerosol; A+AA azithromycin plus antibiotic therapy for aerosol.
