# Supplementary material for: Gut microbiota signatures in cystic fibrosis: Loss of host CFTR function drives the microbiota enterophenotype
Source: PLoS One. 2018 Dec 6;13(12):e0208171. doi: 10.1371/journal.pone.0208171 (PMC6283533; doi:10.1371/journal.pone.0208171)
Supplement: S2 Table — (DOCX) [file pone.0208171.s007.docx]

**S2 Table.** Clinical features of HC subjects: phenomics metadata

| **Patient code** | **Age**  **(years)** | **Gender** | **Z-score/**  **BMI^1^** |
| --- | --- | --- | --- |
| N-06-1 | 6 | F | 0.5 |
| N-06-2 | 6 | M | 1^1^ |
| N-06-4 | 6 | M | 2.5 |
| N-06-5 | 6 | M | -1.5 |
| N-06-6 | 6 | M | 0.4 |
| N-07-3 | 5 | F | 1 |
| N-07-4 | 5 | M | 2.1 |
| N-07-5 | 5 | F | 1.4 |
| N-07-6 | 5 | M | -1.5 |
| N-08-1 | 4 | M | 0.3 |
| N-08-4 | 4 | M | -0.7 |
| N-08-5 | 4 | F | -1.5 |
| N-09-4 | 3 | M | 0.7 |
| N-09-6 | 3 | M | 2 |
| N-09-7 | 3 | F | -1.8 |
| N-09-9 | 3 | F | 0 |
| N-10-1 | 2 | M | -0.5 |
| N-10-2 | 2 | F | 0.8 |
| N-10-3 | 2 | M | 1.6 |
| N-10-4 | 2 | M | -1.4 |
| N-10-5 | 2 | F | -0.9 |
| N-10-6 | 2 | M | 1 |
| N-11-1 | 1 | M | 0 |
| N-11-2 | 1 | M | 0.9 |
| N-11-3 | 1 | M | -1.4 |
| N-11-4 | 1 | F | 1.6 |
| N-11-5 | 1 | F | 0.3 |
| N-11-6 | 1 | M | -1.2 |
| N-11-7 | 1 | F | -0.4 |
| N-11-8 | 1 | M | 0.6 |
| N-11-9 | 1 | M | 0 |

^1^Z-score: W/L for patients under 2 years of age, BMI: for patients older than 2 years of age.
