# Supplementary material for: Gut microbiota signatures in cystic fibrosis: Loss of host CFTR function drives the microbiota enterophenotype
Source: PLoS One. 2018 Dec 6;13(12):e0208171. doi: 10.1371/journal.pone.0208171 (PMC6283533; doi:10.1371/journal.pone.0208171)
Supplement: S3 Table — (DOC) [file pone.0208171.s008.doc]

**S3 Table**. Antibiotic therapy under chronic and exacerbation regimen

|  | **Chronic regimen** | |  | **Exacerbation regimen** |
| --- | --- | --- | --- | --- |
| **Patient code** | **Oral**  (azythromycin 10 mg/kg *per os*) | **Inhaled**  (tobramycin, 300 mg *b.i.d* with on/off regimen) | **Group^1^** | **Oral**  (various, *per os*)^2^ |
| P-06-2 | Not | not | NA | NA |
| P-06-3 | Not | yes | A+AA | Amoxicillin/clavulanic acid (70 mg/kg *per os*) |
| P-06-6 | Yes | yes | A+AA | Amoxicillin/clavulanic acid (70 mg/kg *per os*) |
| P-06-7 | Not | yes | AA | NA |
| P-06-8 | Not | not | NA | Amoxicillin/clavulanic acid (80 mg/kg *per os*) |
| P-07-1 | Yes | not | A+AA | NA |
| P-07-4 | Not | not | NA | NA |
| P-07-5 | Not | not | NA | Cefixime (10 mg/kg *per os*) |
| P-07-6 | Not | yes | AA | NA |
| P-07-7 | Yes | yes | A+AA | Amoxicillin/clavulanic acid (70 mg/kg *per os*) |
| P-07-9 | Not | not | NA | Amoxicillin/clavulanic acid (80 mg/kg *per os*) |
| P-07-10 | Not | not | NA | NA |
| P-08-2 | Not | yes | AA | Amoxicillin/clavulanic acid (80 mg/kg *per os*) |
| P-08-3 | Not | not | NA | NA |
| P-08-4 | Yes | yes | A+AA | Amoxicillin/clavulanic acid (90 mg/kg *per os*) |
| P-08-6 | Not | yes | AA | Amoxicillin/clavulanic acid (80 mg/kg *per os*) |
| P-08-8 | Not | not | NA | Ciprofloxacin (20 mg/kg *per os*) |
| P-09-2 | Not | not | NA | NA |
| P-09-4 | Yes | yes | A+AA | Ciprofloxacin (20 mg/kg *per os*) |
| P-09-6 | Yes | not | A+AA | Amoxicillin/clavulanic acid (90 mg/kg *per os*) |
| P-09-7 | Not | yes | AA | Amoxicillin/clavulanic acid (90 mg/kg *per os*) |
| P-10-2 | Not | yes | AA | NA |
| P-10-4 | Yes | yes | A+AA | Ciprofloxacin (20 mg/kg *per os*) |
| P-10-7 | Not | yes | AA | NA |
| P-10-13 | Not | not | NA | Cefixime (10 mg/kg *per os*) |
| P-11-3 | Not | not | NA | Amoxicillin/clavulanic acid (80 mg/kg *per os*) |
| P-11-4 | Not | not | NA | NA |
| P-11-7 | Not | not | NA | NA |
| P-11-8 | Not | not | NA | NA |
| P-11-9 | Not | not | NA | NA |
| P-11-10 | Not | not | NA | Amoxicillin/clavulanic acid 80 mg/kg *per os* |

^1^Referred to antibiotic chronic regimen; NA, no antibiotic therapy; AA, antibiotic therapy for aerosol; A+AA azithromycin plus antibiotic therapy for aerosol ; ^2^Antibiotic therapy recorded until 2 weeks before stool collection.
