# Supplementary material for: Gut microbiota signatures in cystic fibrosis: Loss of host CFTR function drives the microbiota enterophenotype
Source: PLoS One. 2018 Dec 6;13(12):e0208171. doi: 10.1371/journal.pone.0208171 (PMC6283533; doi:10.1371/journal.pone.0208171)
Supplement: S5 Table — (DOC) [file pone.0208171.s010.doc]

**S5 Table. Number of sequences analyzed, α-diversity indices (ChaoI and Shannon), and estimated sample coverage (ESC) for 16S rRNA amplification from DNA extracted from CF and HC fecal specimens.**

| **Sample** | **No. of OTUs** | **ChaoI** | **Shannon** | **ESC** |
| --- | --- | --- | --- | --- |
| N06.1 | 222 | 263.05 | 5.18 | 0.996 |
| N06.2 | 244 | 285.55 | 4.91 | 0.987 |
| N06.4 | 259 | 306.27 | 5.54 | 0.990 |
| N06.5 | 274 | 325.39 | 5.79 | 0.992 |
| N06.6 | 133 | 152.53 | 4.68 | 0.978 |
| N07.3 | 259 | 319.61 | 5.80 | 0.982 |
| N07.4 | 208 | 246.50 | 5.48 | 0.986 |
| N07.5 | 253 | 324.00 | 5.68 | 0.983 |
| N07.6 | 224 | 279.31 | 5.27 | 0.989 |
| N08.1 | 186 | 221.36 | 4.34 | 0.992 |
| N08.4 | 179 | 208.13 | 4.44 | 0.995 |
| N08.5 | 171 | 190.59 | 4.78 | 0.994 |
| N09.4 | 235 | 266.41 | 5.50 | 0.994 |
| N09.6 | 181 | 222.13 | 4.51 | 0.989 |
| N09.7 | 155 | 214.91 | 3.51 | 0.987 |
| N09.9 | 192 | 243.00 | 4.99 | 0.990 |
| N10.1 | 91 | 128.40 | 3.02 | 0.988 |
| N10.2 | 116 | 174.57 | 3.60 | 0.987 |
| N10.3 | 182 | 224.77 | 4.76 | 0.987 |
| N10.4 | 150 | 222.07 | 4.28 | 0.988 |
| N10.5 | 196 | 272.00 | 5.06 | 0.986 |
| N10.6 | 244 | 309.00 | 5.45 | 0.991 |
| N11.1 | 132 | 181.14 | 2.86 | 0.988 |
| N11.2 | 168 | 195.77 | 5.19 | 0.989 |
| N11.3 | 135 | 162.75 | 3.70 | 0.990 |
| N11.4 | 152 | 190.08 | 4.39 | 0.987 |
| N11.5 | 162 | 193.89 | 4.87 | 0.986 |
| N11.6 | 143 | 210.36 | 4.49 | 0.992 |
| N11.7 | 134 | 183.50 | 4.29 | 0.988 |
| N11.8 | 164 | 201.62 | 3.92 | 0.990 |
| N11.9 | 112 | 135.37 | 3.31 | 0.992 |
| P06.2 | 123 | 158.06 | 4.38 | 0.987 |
| P06.7 | 63 | 72.56 | 2.13 | 0.996 |
| P06.8 | 153 | 186.91 | 4.52 | 0.994 |
| P07.1 | 172 | 191.56 | 4.76 | 0.996 |
| P07.10 | 141 | 213.07 | 3.44 | 0.989 |
| P07.4 | 120 | 161.00 | 4.08 | 0.991 |
| P07.5 | 84 | 102.06 | 2.28 | 0.992 |
| P07.6 | 175 | 249.25 | 4.13 | 0.992 |
| P07.7 | 44 | 59.00 | 2.12 | 0.994 |
| P07.9 | 70 | 85.00 | 3.38 | 0.996 |
| P08.3 | 236 | 275.33 | 5.30 | 0.991 |
| P08.4 | 128 | 149.72 | 3.34 | 0.993 |
| P08.6 | 174 | 193.69 | 4.28 | 0.995 |
| P08.8 | 221 | 252.60 | 4.66 | 0.993 |
| P09.2 | 116 | 151.00 | 3.64 | 0.995 |
| P09.4 | 73 | 92.00 | 2.93 | 0.996 |
| P09.6 | 61 | 74.33 | 3.00 | 0.998 |
| P09.7 | 121 | 173.00 | 2.68 | 0.994 |
| P10.13 | 88 | 106.47 | 2.26 | 0.997 |
| P10.2 | 106 | 160.37 | 3.37 | 0.995 |
| P10.4 | 46 | 56.11 | 1.34 | 0.996 |
| P10.7 | 261 | 296.47 | 5.50 | 0.994 |
| P11.10 | 152 | 193.00 | 4.26 | 0.994 |
| P11.3 | 93 | 114.75 | 3.40 | 0.995 |
| P11.4 | 67 | 121.00 | 1.82 | 0.996 |
| P11.7 | 44 | 55.37 | 4.06 | 0.971 |
| P11.8 | 69 | 84.54 | 3.20 | 0.996 |
| P11.9 | 77 | 106.08 | 1.90 | 0.996 |
