# Supplementary material for: Gut microbiota signatures in cystic fibrosis: Loss of host CFTR function drives the microbiota enterophenotype
Source: PLoS One. 2018 Dec 6;13(12):e0208171. doi: 10.1371/journal.pone.0208171 (PMC6283533; doi:10.1371/journal.pone.0208171)
Supplement: S6 Table — (DOC) [file pone.0208171.s011.doc]

**S6 Table.** Univariate analysis of OTUs, depicting significant differences by the Mann-Whitney U test

| **Mann-Whitney test** | **OIA** | | **IA** | | **p values** |
| --- | --- | --- | --- | --- | --- |
|  | Median | IQR | Median | IQR |  |
| *Clostridium* | 0.027 | 0.186 | 0.513 | 1.878 | 0.016 |
| *Clostridium hiranonis* | 0.175 | 0.369 | 0.870 | 4.910 | 0.025 |
| *Eubacterium* | 0.000 | 0.006 | 0.024 | 0.277 | 0.033 |
| *Faecalibacterium* | 0.010 | 0.027 | 0.152 | 7.541 | 0.028 |
